# Neonatal outcome following norepinephrine versus phenylephrine for treatment of spinal-induced hypotension among women undergoing caesarean section: systematic review and meta-analysis of randomised controlled trials

**DOI:** 10.1186/s13741-026-00701-5

**Published:** 2026-05-28

**Authors:** Tesfaye Asefa Hordofa, Tajera Tageza Ilala, Kurabachew Mengistu

**Affiliations:** 1 Department of Anesthesia, College of Medicine and Health Science, Madda Walabu University, Robe, Oromia Ethiopia; 2 Department of Anesthesia, College of Medicine and Health Science, Hawassa University, Hawassa, Ethiopia

**Keywords:** Caesarean sections, Neonate, Norepinephrine, Phenylephrine, Spinal induced hypotension, Vasopressors

## Abstract

**Background:**

There are pharmacological and non-pharmacological approaches to preventing spinal induced hypotension. In obstetrics, norepinephrine may be an alternative to phenylephrine because it better maintains cardiac output and heart rate. We conducted this systematic review and meta-analysis to identify neonatal outcomes and maternal haemodynamic effects.

**Methods:**

The systematic review of literature searched was conducted through electronic databases such as PubMed, Cochrane Library, and Google Scholar from February 1 to May 30,2025. All of the articles included in the review and meta-analysis were randomised controlled trials, and the populations of the study were normal pregnancies that underwent caesarean section under spinal anesthesia.

**Results:**

A 19 studies of randomised controlled trials involving 2603 participants were included after screening 2446 records. Both prophylactic (MD = 0.17.95%CI: -0.06–0.4, *p* = 0.15) and therapeutic (MD = 0.03,95%CI: -0.18–0.23, *p* = 0.79) administration of norepinephrine showed no statistically significant difference in 1 min Apgar scores compared with phenylephrine. However, norepinephrine was associated with slightly higher 5 min Apgar scores during therapeutic administration compared with prophylactic administration (MD = 0.16,95% CI:0.01–0.31, *p* = 0.04). Norepinephrine revealed that no statistically significant difference in umbilical arterial partial carbon dioxide compared with phenylephrine when administered as bolus, infusion or combined bolus and infusion (MD = -0.98, 95%CI: -3.32–1.35, *p* = 0.41, MD = 0.43,95%CI: -0.54–1.4, *p* = 0.38 and MD = 0.25(-0.42–1.92) respectively. In contrast, norepinephrine significantly increased umbilical arterial bicarbonate level during therapeutic adminstration compared with prophylactic or combined prophylactic or therapeutic administration with phenylephrine (MD = 0.74,95%CI:0.15–1.33.*p* = 0.01). Regarding maternal outcomes,norepinephrine significantly reduced the risk of maternal bradycardia both in prophylactic (RR = 0.52,95%CI:0.34–0.78,*p*-0.02) and in therapeutic administratuin (RR = 0.46, 95%CI:0.29–0.72, *p* = 0.0006) compared with phenylephrine. Furthermore, pooled analysis showed that norepinephrine administration during combined of both prophylactic and therapeutic administration (RR = 0.29,95%CI:0.12–0.65, *p* = 0.003) was associated with lower risk of maternal reflex hypertension than during therapeutic and prophylactic alone compared with phenylephrine.

**Conclusion:**

This systematic review and meta-analysis shows women treated with bolus or infusion, prophylactic or therapeutic administration of norepinephrine have comparable neonatal outcome and maternal haemodynamics with phenylephrine. Therefore, Norepinephrine is an alternative vasopressor for management of spinal induced hypotension and for good neonatal outcome.

**Supplementary Information:**

The online version contains supplementary material available at 10.1186/s13741-026-00701-5.

## Background

Spinal induced hypotension is defined as a reduction in blood pressure more than three-fourths from baseline or a decrease in systolic blood pressure to ≤ 100 mmHg. Due to sympathetic blockage, which leads to vasodilation and increased vascular capacitance, the incidence of spinal induced hypotension can reach up to 75% in patients undergoing caesarean sections. The consequences of spinal hypotension include decreased uterine blood flow, impaired foetal circulation, foetal hypoxia, and maternal bradycardia (Šklebar et al. 2019; Du et al. 2022).

Both pharmacological and non-pharmacological approaches are used to prevent spinal induced hypotension. Positioning (head down tilt/leg elevation, left lateral wedge and manual uterine compression are non-pharmacological strategy, whereas fluids administration, vasopressors, premedication with anti-cholinergic and 5HT3 receptors blockers are pharmacological methods. Among these, vasopressors are still recommended to treat or prevent spinal induced hypotension (Bhat et al. 2024; Mostafa 2023; Partani et al. 2019).

Phenylephrine is a selective α−1agonist with a brief half-life that has been used as a vasopressor in the last ten years for maintaining blood pressure during spinal anaesthesia for caesarean delivery. It is also effective vasopressor for maintaining the foetal acid–base status in healthy women having spinal anaesthesia during caesarean sections; however; it has been associated with decreased cardiac output and baroreceptor-mediated bradycardia (Pan 2024).

Norepinephrine is a potent α- adrenergic agonist with mild β-adrenergic activity, and it is associated with low incidence of bradycardia. But there is no discernible difference between norepinephrine and phenylephrine in cases with reversed post-spinal hypotension with regard to maternal bradycardia. In obstetric practice, norepinephrine may be serve as an alternative to phenylephrine for the management spinal induced hypotension, as it better maintains cardiac output and heart rate (Sharkey et al. 2019; Mohta et al. 2019; Chen et al. 2022).

In both singleton or twin pregnancies, phenylephrine and norepinephrine appear to be equally effective in maintaining maternal haemodynamic stability and neonatal outcome (Chen et al. 2020). Administration of norepinephrine as a bolus following spinal anaesthesia has been shown to reduce the incidence of hypotension and its associated neonatal outcome. However, during the infusion stage, it has no discernible effects on preventing hypotension (Chen et al. 2018; Singh et al. 2022).

The idea that norepinephrine rather than phenylephrine contributes to neonatal foetal acidosis following an elective caesarean delivery is not well supported by the available evidence. On the contrary, norepinephrine may improve placental blood flow and encourage a good neonatal acid base status (Choudhary and Bajaj 2018).

Although phenylephrine remains a first- line drug to prevent spinal induced hypotension, its associated with maternal bradycardia has led to increasing interest in alternative vasopressors. Therefore, this systematic review and meta-analysis aim to explore the effect of phenylephrine or norepinephrine on neonatal outcome following the treatment of spinal induced hypotension and maternal haemodynamics effect.

## Methods

### Protocol

The review was reported in the form of a systematic review and meta-analysis (PRISMA) (Page et al. 2020), and the appraisal was done according to the Grading of Recommendation, Assessment, Development, and Evaluation (GRADE) approach (De Vries et al. 2016). The systematic review research question starts with (PICO). The review was registered to Prospero https://www.crd.york. 1,363,323.

*Participants*: pregnant twins and singletons who underwent a caesarean section under spinal anaesthesia.

*Interventions*: phenylephrine or norepinephrine for the treatment of spinal-induced hypotension.

*Comparator*: comparison of the effect of phenylephrine with norepinephrine on neonatal outcome.

### Outcome

*Primary outcome*: effect of phenylephrine or norepinephrine on neonatal outcome during treatment of spinal-induced hypotension.

*Secondary outcomes*: maternal haemodynamic effects during treatment with phenylephrine or norepinephrine for spinal-induced hypotension.

### Searching strategy

For this systematic review, electronic databases such as PubMed, Cochrane Library, and Google Scholar were used for searches from February 1 to May 30,2025. We searched from PubMed and the Cochrane library by MeSH term and Boolean operator. For example, searching in the PubMed: “neonatal (All fields) “OR” newborn (All fields), neonatal outcome (MeSH) terms “AND” norepinephrine (MeSH terms) “OR” α and β adrenergic agonist (All fields) “AND” phenylephrine (MeSH terms) “OR” selective α 1 agonist (MeSH terms) “AND” spinal induced hypotension (MeSH terms) “OR” “lower systolic blood pressure” (MeSH terms) and “caesarean section" (MeSH terms) (Table S1)). The search methods by Cochrane library was phenylephrine (MeSH terms) “AND” caesarean section (MeSH terms), norepinephrine (MeSH terms) “AND” caesarean section (MeSH terms), “phenylephrine” “AND” norepinephrine (MeSH terms) “AND” Spinal induced hypotension (MeSH terms) (Table S2). From google scholar, randomised controlled trials which much our study was searched up to saturation. The language was restricted to English language. The Mendeley reference 2.138.0 was used to download and remove duplication.

### Eligibility criteria

We used PICO (participants, interventions, comparator, and outcomes) for inclusion or exclusion criteria.

### Inclusion criteria

Population –pregnant women undergoing cesarean section.

Interventions-Prophylaxis and treatment of spinal induced hypotension with norepinephrine versus phenylephrine using s bolus, and infusion based on dosage and time.

### Comparator-phenylephrine with norepinephrine

Outcome-randomized controlled trials that report at least neonatal outcome (Apgar score, Umbilical arterial partial oxygen, umbilical venous partial oxygen, umbilical arterial PH, Umbilical venous PH, base excess, neonatal umbilical partial carbon dioxide, neonatal umbilical venous partial carbon dioxide, neonatal bicarbonate level, and maternal reflex hypertension, bradycardia and hypotension).

Umbilical partial oxygen (UPO2)-it measure the pressure excreted by dissolved oxygen in the blood of the umbilical cord (Higgins 2014).

Umbilical venous partial oxygen (UVPO_2_, 6–31).

- < 6UVPVO_2_- hypoxemia
-  > 31 UVPO_2_- Hyperoxemia

Umbilical arterial partial oxygen (UAPO_2_, 14.4–40).

-  < 14.4 hypoxemia
- > 40 hypoxemia

Umbilical partial carbon dioxide (UPCO_2_)-the partial pressure of carbon dioxide in umbilical cord (Cai et al. 2022).

Umbilical venous partial carbon dioxide (UVPCO_2_, 29–54).

 

- < 29 hypocarbia
-  > 54 hypercarbia

Umbilical arterial partial carbon dioxide (UAPCO_2_, 42–74).

-  < 42 -hypocarbia
-  > 74-hypercarbia

PH-it Determine acidity level of blood within umbilical cord (Olofsson 2023).

Umbilical venous PH (UVPH) (7.25–7.45).

- PH < 7.25 feotal acidosis
- PH > 7.45 feotal alkalemia

Umbilical arterial PH (UAPH) (7.18–7.38).

 

- < 7.18 Feotal acidosis
-  > 7.38- feotal alkalemia

Base excess (BE): the amount of base needed to restore normal PH at 37°c (Cai et al. 2022).

- BE < −2 Metabolic acidosis
- BE > 2Metabolic alkalosis

Reflex hypertension-A phenomenon where body’s normal blood pressure exacerbates ongoing state of high blood pressure.

Bradycardia- Heart rate that is excessively slow or less than 60 beats per minute.

Exclusion criteria: articles other than randomised controlled trials, studies not related to neonatal outcome or maternal haemodynamic effects, lack of authorship, animal studies and articles published in languages other than English.

### Data collection and analysis

#### Selection of studies

The articles were gathered by two authors and then added to an Excel spreadsheet. At first T.A. and K.M. were using mendeley reference to exclude the duplication. T.T evaluate whether the articles to meet the topic based on abstract and titles. Each of authors independently retrieved and checked the screened data based on authors, sample size, country, year of publication, study design and methods of administrations.

### Data extraction

We collected data on the review of articles depending on the characteristics of the study, methodology, and outcome. All included articles were randomised controlled trials. We used an Excel spreadsheet to extract the necessary information and review manager software (version 5.4.1) for the analysis of the data (Table 1). Three authors were involved in data extraction.

**Table 1:** Characteristics of study of neonatal outcome following norepinephrine versus phenylephrine for treatment of spinal induced hypotension among women undergoing caesarean section:SRMA,2025

Authors	study design	Sample size	Country	Type of surgery and pregnancy	Intervention	Outcome
Liu et al. (2022)	RCT	78	China	Elective/singleton	0.54 mcg/kg/min infusion of phenylephrine and 0.08mcg/kg/min infusion of norepinephrine and 2mcg/kg/min infusion of metarminol as prophylaxis	Infusion of phenylephrine and norepinephrine were not significant difference on neonatal acid base status
Mohta et al. (2021)	RCT	100	India	Emergency/singleton	100 mcg of intravenous phenylephrine versus 8mcg of intravenous norepinephrine for treatment of spinal induced hypotension	Intravenous administration of 100mcg of phenylephrine and 8mcg of norepinephrine have similar neonatal outcome for treatment neonatal outcome for treatment
Nguyen et al. (2023)	RCT	220	Vietnam	Elective/singleton	6 mcg bolus of norepinephrine versus 100mcg bolus of phenylephrine	Bolus intravenous administration of norepinephrine and phenylephrine were equally effective in treating spinal induced hypotension and similar neonatal and maternal outcome
Ravichandrane et al. (2023)	RCT	130	India	Elective/singleton	5mcg/min of norepinephrine infusion versus 25mcg/min of phenylephrine infusion for spinal induced hypotension treatment	Both medication were equally prevented post spinal induced hypotension if given as prophylactic infusion
Puthenveettil et al. (2019)	RCT	50	India	Elective/Singleton	50 mcg bolus phenylephrine versus 4mcg bolus norepinephrine as therapeutic norepinephrine as therapeutic	Intermittent bolus of norepinephrine were effective for management of spinal induced hypotension Neonatal outcome were similar in both group
J.P.Tiwari et al. (2022)	RCT	**126**	India	Elective/singleton	50 mcg bolus of phenylephrine versus 4mcg of bolus of norepinephrine as therapeutic	Intermittent bolus of norepinephrine were effective for management of spinal induced hypotension
Woo jin Cho et al. (2020)	RCT	56	Korea	Elective/singleton	100 mcg/ml bolus of phenylephrine versus 5mcg/ml of bolus norepinephrine as therapeutic	Intermittent bolus intravenous of norepinephrine were effective for management of spinal induced hypotension with maintaining of cardiac output and stroke volume
Sharkey Aidan M. (2019)	RCT	112	Canada	Elective/Singleton	100mcg/ml bolus of phenylephrine versus 6mcg/ml of norepinephrine as prophylactic or treatment	Intermittent bolus intravenous of norepinephrine were superior to phenylephrine to maintain haemodynamic stability
K.Theodoraki et al. (2020)	RCT	82	Greece	Elective/singleton	Fixed rate infusion of norepinephrine 4mcg/min versus fixed rate infusion of phenylephrine prophylactic	Fixed rate infusion of norepinephrine is similar to fixed rate infusion of phenylephrine for management of spinal induced hypotension
Guo et al. (2022)	RCT	138	China	Elective/singleton	0.625 mg/kg/min infusion of phenylephrine versus 0.05mcg/kg/min of norepinephrine infusion as prophylactic	No difference on incidence of hypotension and neonatal outcome if both medication is giving as prophylactic
Ngan Kee et al. (2020)	RCT	664	China	Elective or Emergency	6mcg/ml bolus or infusion of norepinephrine versus 100mcg/ml bolus or infusion of	Norepinephrine has good safety for neonatal given either as prophylactic or therapeutic phenylephrine
Du et al. (2022)	RCT	62	China	Elective/Twin	6mcg/ml of norepinephrine infusion versus 75mcg/ml of phenylephrine infusion as prophylactic	Fixed rate infusion of norepinephrine and phenylephrine are maintaining blood pressure in twin pregnancy
Rai et al. (2022)	RCT	90	India	Elective/singleton	7.5mcg of bolus norepinephrine versus 100mcg of bolus phenylephrine as therapeutic	Normotensive patients who take bolus dose of phenylephrine and norepinephrine have comparable neonatal umbilical cord blood gas analysis and Apgar score but norepinephrine has lower bradycardia and reactive hypertension than phenylephrine
Ali and Bajaj et al. (2023)	RCT	100	India	Elective/Emergency-	0.1mcg/kg/min of phenylephrine infusion versus 0.05mcg/kg/min of norepinephrine infusion as prophylactic	Norepinephrine is better maintaining of hemodynamic stability when giving as prophylactic and its advantageous in pregnancy induced hypertension
Eskandr AM et al. (2020)	RCT	75	Egypt	Elective/singleton	0.1mcg/kg/min of phenylephrine infusion versus 0.05mcg/kg/min of norepinephrine infusion as prophylactic	Prophylactic infusion of phenylephrine and norepinephrine can be successfully used to prevent post spinal hypotension and foetal wellbeing than ephedrine
Renu et al. (2022)	RCT	80	India	Elective/singleton	10mcg of bolus norepinephrine versus 50mcg of bolus phenylephrine as prophylactic	The efficacy in rescue of norepinephrine is similar to phenylephrine in preventing of spinal induced hypotension
Oliver B et al. (2023)	RCT	124	France	Elective/both	0.05mcg/kg/min of norepinephrine infusion versus 0.5 mcg/kg/min of phenylephrine infusion as prophylactic	Manual adjusted infusion of both medication were effective for maintaining blood pressure but norepinephrine infusion has higher cardiac index
Saha et al. (2024)	RCT	95	India	Elective/Singleton	5mcg bolus norepinephrine versus 50 mcg bolus phenylephrine as therapeutic	neonatal and maternal outcome were similar for both norepinephrine and phenylephrine bolus regimen. So norepinephrine is an alternative to phenylephrine for treatment of post spinal hypotension
Singh et al. (2021)	RCT	105	India	Elective/singleton	5mcg/min infusion of norepinephrine versus 100mcg/min infusion of phenylephrine as prophylactic	Prophylactic infusion of norepinephrine has higher base excess than phenylephrine but has comparable foetal acidosis. Phenylephrine resulted in higher bradycardia than norepinephrine

### Data analysis

Review manager version 5.4.1 software was used for data analysis. We used mean difference (MD) with 95% CI for continuous data and relative risk (RR) with 95% CI for dichotomous data. Heterogeneity was assessed by using I^2^ and chi^2^_._ A *p* value < 0.05 considered as heterogeneity from Chi –square_._ Eggers test with funnel plot symmetry was used to check publication bias. Eggers test was conducted by using STATA version 16. We used random effect model for I^2^ ≥ 50%, geographical heterogeneity, and greater than three study and a fixed effect model for I^2^ ≤ 30% and less than three studies. Subgroup and pairwise group were analysed by using a forest plot. Subgroup analysed was performed to explore source of high heterogeneity. For both maternal and neonatal outcome, subgroup analysis was conducted based on timing of administration (prophylactic versus therapeutic), dose response (bolus versus infusion; high dose versus low dose), type of pregnancy (singleton versus twin pregnancy), and type of procedure (elective versus emergence).

### Quality of assessment and risk of assessment

The included studies were assessed with the Cochrane Collaboration’s tool for assessing the risk of bias for RCTs (Cumpston et al. 2022). Quality of evidence was assessed using the GRADE approach by using risk of bias, inconsistency, indirectness, imprecision and publication bias (*Clinical Epidemiology and Global health 25 *(2024) (Epidemiology 2024). Risk of bias was assessed by using Review manager software version 5.4.1.

## Results

### Trial

**Fig. 1:**
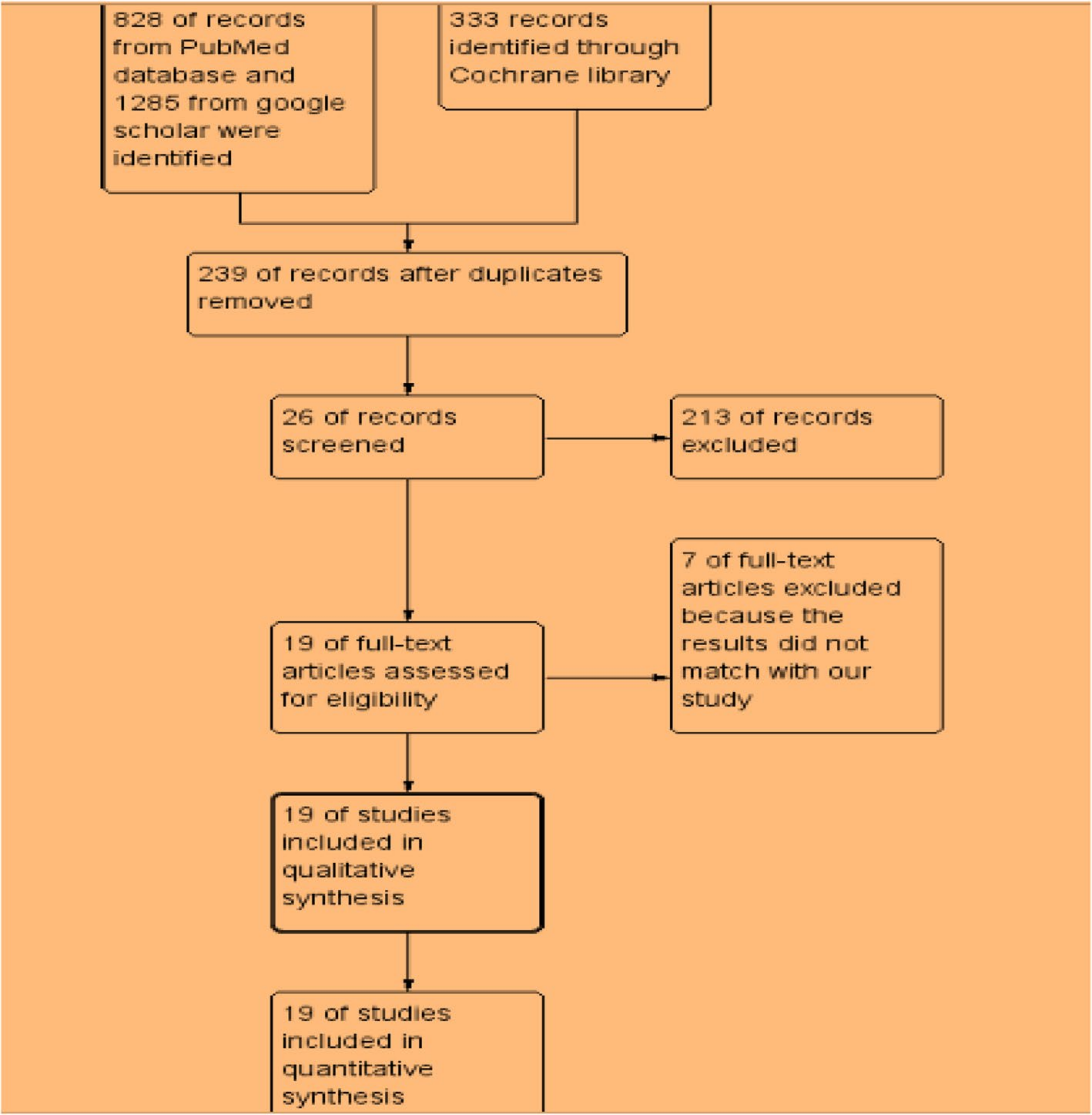
PRISMA flow chart of neonatal outcome following norepinephrine versus phenylephrine for treatment of spinal induced hypotension among women undergoing cesarean sections: SRMA, 2025

For this review, 828 studies from PubMed, 1285 studies from Google Scholar, and 333 studies from the Cochrane Library were collected. After being restricted by year with full text, the total study was finally 239. After duplication removed and screening, 19 studies were included in this systematic review (Fig. 1). The mode of drug administration and rate of infusions for the study were considered again. The haemodynamic base line, height, and weight of the two groups (who take norepinephrine and phenylephrine) were comparable (Cho 2020; Cv 2023; Mukherjee and Bagga 2021).

### Characteristics of study

The characteristics of included studies were summarised in (Table 1). From the gathered articles, 16 were published in Asia countries, two articles were in Europe and one was in Africa**.**Patients included in the eligibility criteria were in normal singleton and twin pregnancies, either undergoing elective or emergency caesarean delivery under spinal anesthesia. The patients were pre-medicated with metoclopramide (10 mg), ranitidine (50 mg)and ondansetron 4 mg for 1 h before surgery (Mohta et al. 2019; Cv 2023; Eskandr 2020) and pre-hydrated and co-hydrated with linger lactate(15 ml/kg) (Kee et al. 2020; Rai 2022; Article 2019). All patients were in the left lateral position immediately after entering the operating room, and spinal anesthesia was administered using bupivacaine with fentanyl using a 25-gauge spinal needle (Sharkey et al. 2019; Double-blinded 2022).

### Risk of bias assessment

**Fig. 2:**
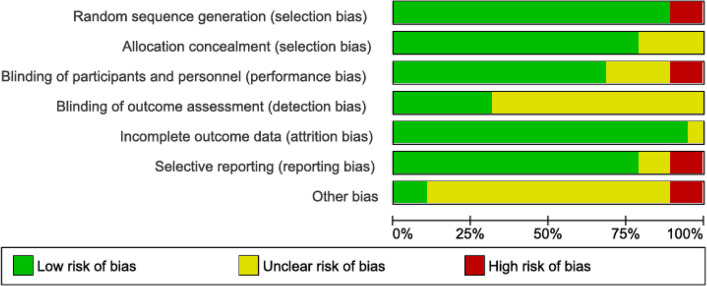
Risk of bias graph of neonatal outcome following norepinephrine versus phenylephrine for treatment of spinal induced hypotension among women undergoing cesarean sections: SRMA, 20,025

**Fig. 3:**
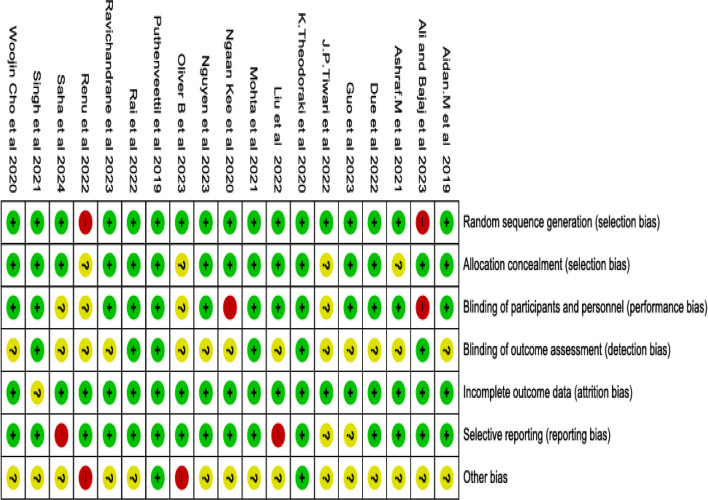
Risk of bias summary of neonatal outcome following norepinephrine versus phenylephrine for treatment of spinal induced hypotension among women undergoing cesarean sections: SRMA, 2025

We assessed the risk of bias based on a random sequence of generation, allocation of context, blinding of personnel and participants, blinding of outcome assessment, incomplete data, reporting bias and other bias (Fig. 2). The risk of bias was explained by a graph using software review manager version 5.4.1). The risk of bias was categorized as high, low and unclear by using graph summary (Fig. 3). The risk of bias tables and appraisal trials was determined by using the Cochrane Collaboration tool.

### Quality of assessment

The quality of study was assessed by using Cochrane collaboration tool for the included study. The certainty of evidence was assessed by using GRADE approach. It is categorized as low, moderate and high evidence after assessed publication bias, risk of bias, imprecision, indirectness, and inconsistency (Table 2). Overall five studies were rated as having low quality evidence (Cho 2020; Cv 2023; Eskandr 2020; Guo et al. 2022), from this two studies have high risk of bias, three studies are at a risk of publication bias and one article has a high variability, five studies have moderate quality of evidence (Sharkey et al. 2019; Kee et al. 2020; Article 2019, 2024; Belin et al. 2023) and nine studies have a high level of quality (Mukherjee and Bagga 2021; Rai 2022; Liu et al. 2022; Mohta et al. 2022; Theodoraki et al. 2020; Tiwari et al. 2022).

**Table 2:** Level of quality assessment of neonatal outcome following norepinephrine versus phenylephrine for treatment of spinal induced hypotension among women undergoing cesarean section: SRMA, 2025

Authors	Study design	Risk of bias	Inconsistency	Indirectness	Imprecision	Publication bias	level of quality (GRADE)
Liu et al. (2022)	RCT	Not serious	Not serious	Not serious	Not serious	Undetected	High
Mohta et al. (2021)	RCT	Not serious	Not serious	Not serious	Not serious	Undetected	High
Nguyen et al. (2023)	RCT	Not serious	Not serious	Not serious	Not serious	Undetected	High
Ravichandrane et al. (2023)	RCT	Not serious	Not serious	Not serious	Not serious	Undetected	High
Puthenveettil et al. (2019)	RCT	Not serious	Not serious	Not serious	Serious	suspected	Moderate
J.P.Tiwari et al. (2022)	RCT	Not serious	Not serious	Not serious	Not serious	Undetected	High
Woo jjin Cho et al. (2020)	RCT	Not serious	Not serious	Serious	Serious	suspected	Very low
Sharkey Aidan M. et al. (2019)	RCT	Not serious	Not serious	Not serious	Not serious	suspected	Moderate
K.Theodoraki et al. (2020)	RCT	Not serious	Not serious	Not serious	Not serious	Undetected	High
Guo et al. (2022)	RCT	Serious	Not serious	Not serious	Not serious	suspected	Low
Ngaan Kee et al. (2020)	RCT	Not serious	Not serious	Not serious	Not serious	suspected	Moderate
Due et al. (2022)	RCT	Not serious	Not serious	Not serious	Not serious	Undetected	High
Rai et al. (2022)	RCT	Not serious	Not serious	Not serious	Not serious	Undetected	High
Ali and Bajaj (2023)	RCT	Serious	Not serious	Not serious	Not serious	suspected	Low
Eskandr AM et al. (2020)	RCT	Not serious	Not serious	Not serious	Serious	suspected	Low
Renu et al. (2022)	RCT	Not serious	Not serious	Not serious	Not serious	suspected	Low
Oliver B et al. (2023)	RCT	Not serious	Not serious	Not serious	Not serious	suspected	Moderate
Saha et al. (2024)	RCT	Not serious	Not serious	Not serious	Not serious	suspected	Moderate
Singh et al. (2021)	RCT	Not serious	Not serious	Not serious	Not serious	Undetected	High

*RCT* Randomized controlled trial

### Sensitivity analysis

Sensitivity analysis was conducted by using leave-one-out methods and excluding high risk of bias for assessing robustness of findings. Leave one-out methods were assessing bolus versus infusion on umbilical venous partial carbon dioxide, prophylactic versus therapeutic on umbilical base excess, umbilical arterial base excess during infusion versus bolus, umbilical arterial bicarbonate level during prophylaxis and maternal reflex hypertension during prophylactic. Leave –one –out approach demonstrated that there is a consistent pooled effect regarding to umbilical arterial bicarbonate level during prophylactic versus therapeutic (MD = 0.95,95% CI:0.4–1.5, *p* = 0.0008, I^2^ = 0%).After sensitivity analysis heterogeneity was substantially reduced (Tau^2^ = 0.07, chi^2^ = 6.49, df = 5, *p* = 0.26; I^2^ = 23%).The pooled analysis showed a statistically significant overall effect (Z = 2.94, *p* = 0.003). Similarly, the pooled effect of maternal reflex hypertension during bolus administration versus infusion revealed that no robustness of finding (RR = 0.83,95% CI:0.34–2.06. *p* = 0.69, I^2^ = 0%). The heterogeneity was reduced after sequential analysis (Tau^2^ = 0.00, chi^2^ = 5.36, df = 6, *p* = 0.5, I^2^ = 0%) but no significant overall effect (Z = 1.26, *p*= 0.21). The removing of a single study resulted in change the overall pooled effect for umbilical venous partial carbon dioxide during bolus administration, umbilical arterial base excess during prophylactic administration, umbilical arterial base excess during infusion, umbilical arterial PH during therapeutic and bolus administration (Table S3). Removing of high risk of bias (Cv 2023; Kee et al. 2020; Liu et al. 2022) can be affect overall pooled effects.

### Subgroup analysis

A post hoc subgroup analysis was performed to evaluate the effects of timing of administration, dosage, methods of administration, type of procedure, type of pregnancy and high versus low dose regimens on neonatal outcome and maternal haemodynamic effects (Table 3). A subgroup analysis of Apgar score at 1 and 5 min showed no statistically significant subgroup effects according to prophylactic versus therapeutic administration or bolus versus infusion methods. Similarly, no statistically significant differences were observed between norepinephrine and phenylephrine for neonatal Apgar scores either 1 or 5 min across all subgroups. Regarding umbilical venous partial carbon dioxide norepinephrine significantly reduced UVPCO_2_ compared with phenylephrine when administered therapeutically rather than prophylactically or combined prophylactic and therapeutic regimens (MD = −2.46,95% CI: −4.52- −0.4, *p* = 0.02). However, the subgroup difference was not statistically significant (Chi^2^ = 4.75, df = 2, *p* = 0.09, I^2^ = 57.9%). For umbilical venous PH norepinephrine was associated with significantly higher UVPH compared with phenylephrine when administrated combined prophylactic and therapeutic regimens rather than prophylactic or therapeutic administration alone (MD = 0.01,95%CI: 0.00–0.01, *p* = 0.009). Nevertheless, no statistically significant subgroup effect was identified (Chi^2^ = 4.73, df = 2, *p* = 0.09, I^2^ = 57.7%). Regarding to maternal haemodynamic effects, norepinephrine leads to bradycardia more than phenylephrine when administered as a both bolus (RR = 0.49,95%CI:0.35–0.67, *p* < 0.0001) and infusion (RR = 0.46,95%CI:0.29–0.71. *p* = 0.0006). No statistically significant difference was identified, although heterogeneity was substantially reduced (Chi^2^ = 0.06, df = 1, *p* = 0.81, I^2^ = 0%).

**Table 3:** Summary of subgroup analysis of neonatal outcome following norepinephrine versus phenylephrine for treatment of spinal induced hypotension among women undergoing caesarean section: SRMA,2025

Subgroup	Comparison group	Sample size	Numbers of study	Effect size(95%CI)	*P* value	Subgroup difference	Overall pooled effect	Inter group I^2^(%)
Apgar score at 1 min (high/low)	Infusion	424	5	MD = 0.1(−0.12–0.32)	0.37	Chi^2^ = 0.04,df = 1,p = 0.84	MD = 0.08 (−0.05–0.22)	0
	bolus	553	6	MD = 0.07(−0.1–0.25)	0.65	I^2^ = 0%		0
Apgar score at 5 min (infusion/bolus)	Infusion	383	5	MD = 0.00 (−0.17–0.17)	1	Chi^2^ = 0.00,df = 1,p = 0.96	MD = −0.001 (−0.07–0.06)	0
	bolus	553	6	MD = −0.00 (−0.07–0.06)	0.9	i^2^ = 0%		0
Umbilical arterial PCO2(methods of administration	prophylactic	406	5	MD = 0.3(−0.58–1.18)	0.51	Chi^2^ = 0.89	MD = 0.06 (−0.41–0.52)	0
	therapeutic	384	4	MD = −0.29 (−2.39–1.82)	0.79	,df = 2,p = 0.64,I^2^ = 0%		65
	combined of therapeutic and prophylactic	806	2	MD = −0.96 (−3.70–1.78)	0.49			
Umbilical arterial PCO2 (high/low bolus dose)	at high bolus dose	366	4	MD = −0.7(−2.65–1.26)	0.48	Chi^2^ = 0.03,df = 1,p = 0.86	MD = −0.62 (−2.26 −1.02)	59
	at low bolus dose	95	1	MD = −0.35(−3,74–3.04)	0.84	I^2^ = 0%		0
Umbilical arterial PO2 (methods of administration	prophylactic	753	8	MD = −0.43 (−1.4–0.54)	0.39	Chi^2^ = 0.25,df = 2,p = 0.88	MD = −0.39 (−1.11–0.33)	0
	therapeutic	600	6	MD = −0.55 (−1.89–0.8)	0.43	I^2^ = 0%		0
	combined of therapeutic and prophylactic	776	2	MD = 0.00 (−1.74–1.74)	1			0
Umbilical arterial PCO2(high/low infusion)	High infusion	232	3	MD = 2.08 (−0.15–4.31)	0.07	Chi^2^ = 2.99,df = 1,p = 0.08	MD = 0.12(−0.1–0.34)	
	low infusion	304	3	MD = 0.1(−0.09–0.3)	0.31	I^2^ = 66.6%		4
Umbilical arterial PO2(high/low bolus)	High bolus	1202	6	MD = −0.3(−1.6–1.01)	0.66	Chi^2^ = 1.58,df = 2,p = 0.45	MD = −0.39 (−1.11–0.33)	0
	low bolus	788	2	MD = 0.84(−1.34–3.02)	0.45	I^2^ = 0%		0
Umbilical venous PCO2 (bolus/infusion)	bolus	252		MD = 1.64 (−5.96–9.25)	0.67	Chi^2^ = 0.19,df = 2,p = 0.91	MD = 0.61 (−1.71–2.93)	94
	infusion	134	2	MD = −0.08(−1,53–1.37)	0.94	I^2^ = 0%		0
	combined of infusion and bolus	694	1	MD = 0.00(−1–1)	1			
Umbilical venous PCO2 (methods of administration	prophylactic	134	2	MD = −0.08(−1,53–1.37)	0.91	Chi^2^ = 4.75,df = 2,p = 0.09	MD = 0.61 (−1.71–2.93)	0
	therapeutic	140	2	MD = −2.46(−4.52- −0.4)	0.02	I^2^ = 0%		0
	combined therapeutic and prophylactic	806	2	MD = 4.35(−4.47–13.16)	0.33			96
Umbilical venous PCO2(high/low bolus dose)	high bolus dose	202	2	MD = 4(−5.96–13.97)	0.43	Chi^2^ = 5.66,df = 3,p = 0.13	MD = 53 (1.5–1.65)	92
	low bolus	50	1	MD = −2.84(−5.18- −0.5)	0.02	I^2^ = 47%		
Umbilical arterial HCO3 Level (infusion/bolus)	infusion	202	3	MD = 0.33 (−0.92–1.56)	0.6	0.04,df = 1,p = 0.84,I^2^ = 0%	MD = 0.43 (−0.19–1.05)	84
	bolus	417	4	MD = 0.48(−0.04–0.99)	0.07			15
Umbilical arterial HCO3 Level(high/low bolus)	High bolus dose	986	4	MD = 0.01(−0.02–0.03)	0.51	Chi^2^ = 0.00,df = 1,p = 1,I^2^ = 0%	MD = 0.1(−0.01–0.03)	79
	low bolus dose	44	1	MD = 0.02(−4.3–4.34)	0.99			
Umbilical venous HCO3 (methods of administration	prophylactic	172	2	MD = −3.28(−0.42–6.97	0.08	Chi^2^ = 2.41,df = 1,p = 0.12	MD = 2.24(−0.59–5.07)	
	therapeutic	52	1	MD = 0.27(−0.62–1.16)	0.55	I^2^ = 58.5%		
Umbilical venous HCO3 (bolus/infusion	bolus	164	2	MD = −0.39(−1.63–0.85)	0.54	Chi^2^ = 3.4,df = 1,p = 0.07	MD = 1.39(−0.88–3.65)	78
	infusion	172	2	MD = 3.28(−0.42–6.97	0.08	I^2^ = 70.6%		92
Umbilical arterial base excess (methods of administration	prophylactic	414	4	MD = 0.36(−0.56–1.28)	0.44	Chi^2^ = 0.29,df = 2,p = 0.87	MD = 0.19(−0.23–0.61)	74
	therapeutic	708	2	MD = 0.13(−0.24–0.5)	0.49	I^2^ = 0%		0
	combined	112	1	MD = 0.3(−0.61–1.21)	0.52			
Umbilical arterial base excess (bolus/infusion/	bolus	156	2	Md = 0.06(−0.52–0.65)	0.83	Chi2 = 0.16,df = 2,p = 0.92	MD = 0.06 (−0.32–0.44)	0
combined)	infusion	362	3	MD = 0.08(−0.87–1.02)	0.87	I^2^ = 0%		70
	combined	664	1	MD = 0.2(−0.22–0.62)	0.35			
Umbilical arterial base excess (high/low infusion	high infusion dose	238	2	MD = 0.46(−1.2–2.12)	0.59	Chi^2^ = 1,df = 1,p = 0.32	MD = 0.08(−0.87–1.02)	82
	low infusion dose	124	1	MD = −0.5(−1.39–0.39)	0.27	I^2^ = 0.1%		
Umbilical venous base excess (methods of administration	prophylactic	716	2	MD = 0.21(−0.15–0.57)	0.25	Chi^2^ = 6.96,df = 2,p = 0.03	MD = 0.34(−0.23–0.91)	0
	therapeutic	112	1	MD = −0.25(−1.07–0.57)	0.55	I^2^ = 71.3%		
	combined therapeutic and prophylactic	82	1	MD = 1.5(0.45–2.55)	0.005			
Umbilical arterial PH (infusion/bolus)	infusion	416	4	MD = 0.00(−0.01–0.01)	1	Chi^2^ = 0.39,df = 1,p = 0.53	MD = 0.01(−0.01–0.02)	0
	bolus	1156	6	MD = 0.01(−0.01–0.03)	0.47	I^2^ = 0%		65
UAPH (high/low bolus)	high bolus dose	986	4	MD = 0.01(−0.02–0.03)	0.51	Chi^2^ = 0.00,df = 1,p = 1	MD = 0.01(−0.01–0.03)	79
	low bolus dose	44	1	MD = 0.02(−4.3–4.34)	0.99	I^2^ = 0%		
Umbilical venous PH (Infusion/bolus)	infusion	277	3	MD = 0.01(−0.01–02)	0.17	Chi^2^0.56,df = 1,p = 0.46	MD = 0.01(0.00–0.01)	0
	bolus	544	4	MD = 0.01(−0.02–0.02)	0.85	I^2^ = 0%		46
UVPH(methods of administration)	prophylactic	277	3	MD = 0.01(−0.00–0.02)	0.17	Chi^2^ = 4.73,df = 2,p = 0.09	MD = 0.01(−0.00–0.02)	0
	therapeutic	168	2	MD = −0.03(−0.06–0.00)	0.09	I^2^ = 57.7%	MD = −0.03(−0.06–0.00)	0
	combined prophylactic and therapeutic	776	2	MD = 0.01(0.00–0.01)	0.009		MD = 0.01(0.00–0.01)	0
UVPH(High/low bolus)	High bolus	168	2	MD = −0.03(−0.06–0.00)	0.09	Chi2 = 4.73,df = 2,p = 0.09	MD = 0.01(−0.00–0.02)	0
	low bolus	776	2	MD = 0.01(0.00–0.01)	0.009	I2 = 57.7%		0
Maternal bradycardia(bolus/infusion)	bolus	1328	8	RR = 0.49(0.35–0.67)	< 0.0001	Chi^2^ = 0.1,df = 1,p = 0.76	RR = 0.52(0.41–0.65)	22
	infusion	632	6	RR = 0.53(0.35–0.81)	0.003	I^2^ = 0%		16
Maternal bradycardia(pregnancy type)	singleton	1020	10	RR = 0.41(0.28–0.59)	< 0.00001	Chi^2^ = 6.69,df = 4,p = 0.1	RR = 0.52(0.41–0.65)	0
	Twin	62	1	RR = 0.35(0.17–0.71)	0.003	I^2^ = 40.2%		
	Both	124	1	RR = 0.83(0.39–1.79)	0.64			
Maternal bradycardia (high versus low bolus)	high bolus dose	1078	5	RR = 0.41(0.41–0.99)	0.05	Chi^2^ = 0.13,df = 2,p = 0.94	RR = 0.49(0.39–0.63)	19
	low bolus	250	3	RR = 0.49(0.37–0.72)	0.0003	I^2^ = 0%		34
Maternal bradycardia(type of procedure)	Elective	1144	12	RR = 0.39(0.29–0.59)	< 0.00001	Chi^2^ = 5.38,df = 2,p = 0.07	RR = 0.49(0.39–0.63)	0
	Emergency	90	1	RR = 0.59(0.3–1.14)	0.12	I^2^ = 62.8%		
	combined elective &emergency	664	1	RR = 0.63(0.5–0.79)	< 0.0001			
	High dose infusion	216	2	RR = 0.28(0.12–0.7)	0.04	Chi^2^ = 2.22,df = 1,p = 0.14	RR = 0.53(0.35–0.81)	19
	low dose infusion	416	4	RR = 0.61(0.38–0.98)	0.006	I^2^ = 55%		0
Maternal reflex hypertension (bolus versus	bolus	246	3	RR = 0.53(0.21–1.3)	0.16	Chi^2^ = 0.56,df = 1,p = 0.45	RR = 0.66(0.41–1.09)	43
infusion)	infusion	532	5	RR = 0.8(0.44–1.45)	0.46	I^2^ = 0%		15
Maternal reflex hypertension(type of pregnancy	singleton	592	7	RR = 0.71(0.31–1.67)	0.44	Chi^2^ = 0.82,df = 2,p = 0.66	RR = 0.66(0.41–1.09)	40
	Twin	62	1	RR = 0.59(0.32–1.07)	0.08	I^2^ = 0%		
	both twin and singleton	124	1	RR = 1(0.37–2.68)	1			
Maternal reflex hypertension(high versus	high bolus dose	90	1	RR = 2(0.19–21.28)	0.57	Chi^2^ = 1.4,df = 1,p = 0.24	RR = 0.53(0.21–1.3)	
low bolus	low bolus dose	156	2	RR = 0.43(0.18–1.07)	0.07	I^2^ = 28.5%		50
Maternal reflex hypertension (high infusion	high infusion	278	3	RR = 0.62(0.36–1,09)	0.1	Chi^2^ = 1.11,df = 1,p = 0.29	RR = 0.8(0.44–1.45)	0
versus low infusion)	low infusion	254	2	RR = 1.63(0.3–8.88)	0.57	I^2^ = 9.7%		37
Maternal reflex hypertension(type of pocedure-	Elective	644	7	RR = 0.64(0.34–1.21)	0.17	Chi2 = 0.83,df = 1,p = 0.36	RR = 0.68(0.37–1.25)	40
re		90	1	RR = 2(0.19–21.28)	0.57	I2 = 0%		

### Synthesis of analysis

#### Neonatal outcome

**Fig. 4:**
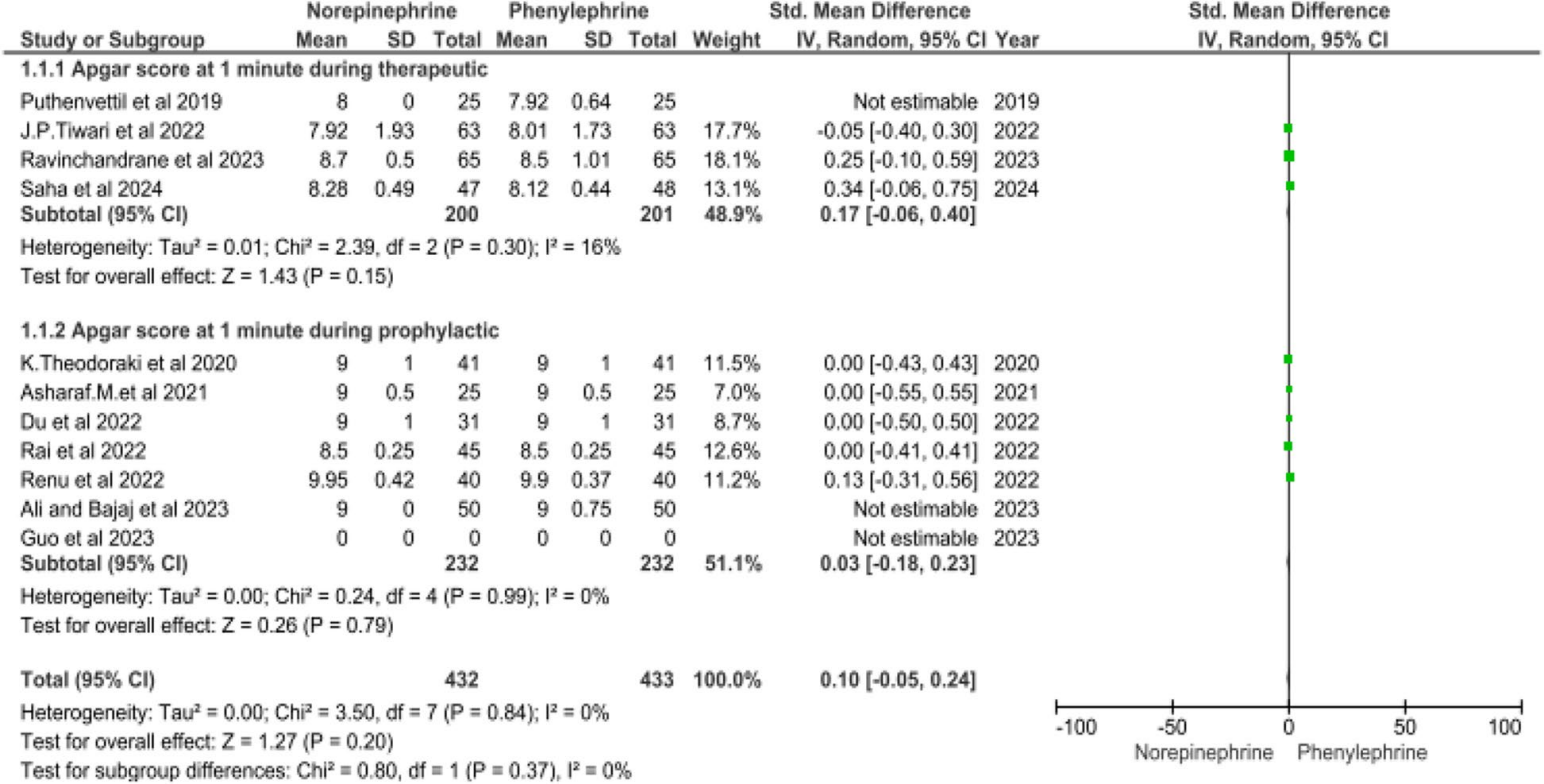
Forest plot shows comparison of therapeutic versus prophylactic administration of norepinephrine and phenylephrine on Apgar score < 7 at 1 min among women undergoing caesarean section:: SRMA, 2025

**Fig. 5:**
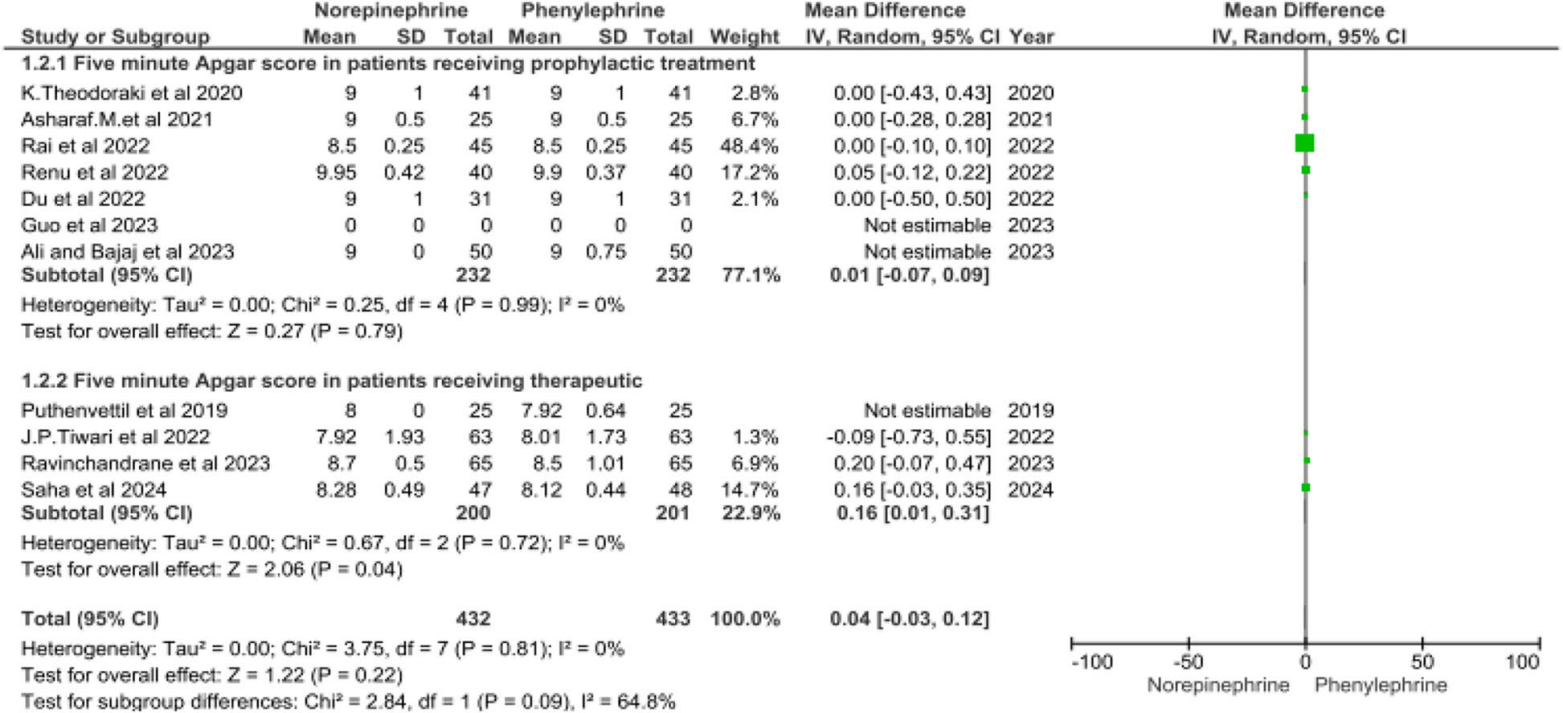
Forest plot shows comparison of therapeutic versus prophylactic administration of norepinephrine and phenylephrine on Apgar score < 7 at 5 min among women undergoing caesarean section: SRMA, 2025

**Fig. 6:**
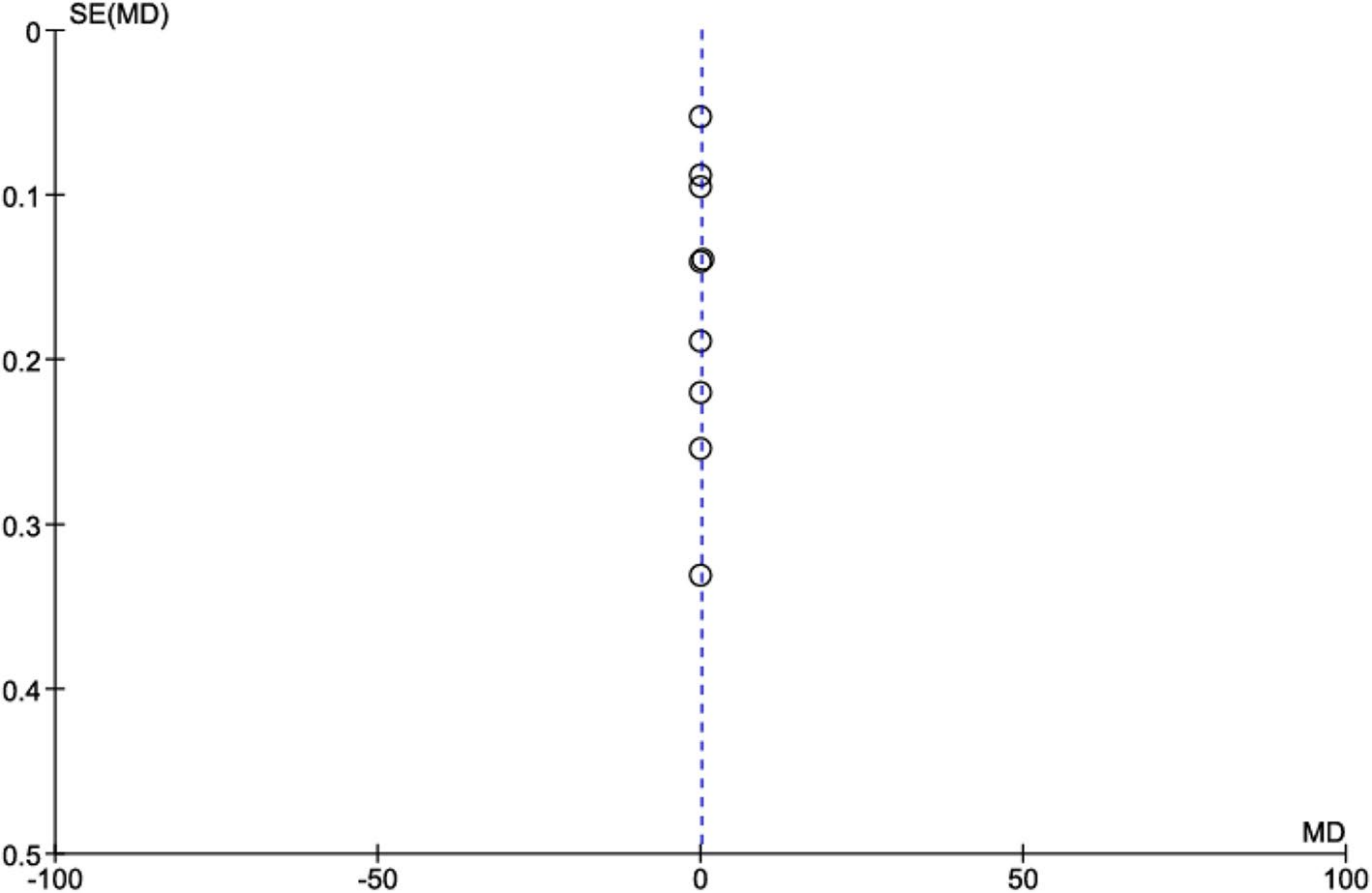
Funnel plot shows Apgar score < 7 at one minute following norepinephrine versus phenylephrine for treatment of spinal induced hypotension among women undergoing cesarean section: SRMA, 2025

**Fig. 7:**
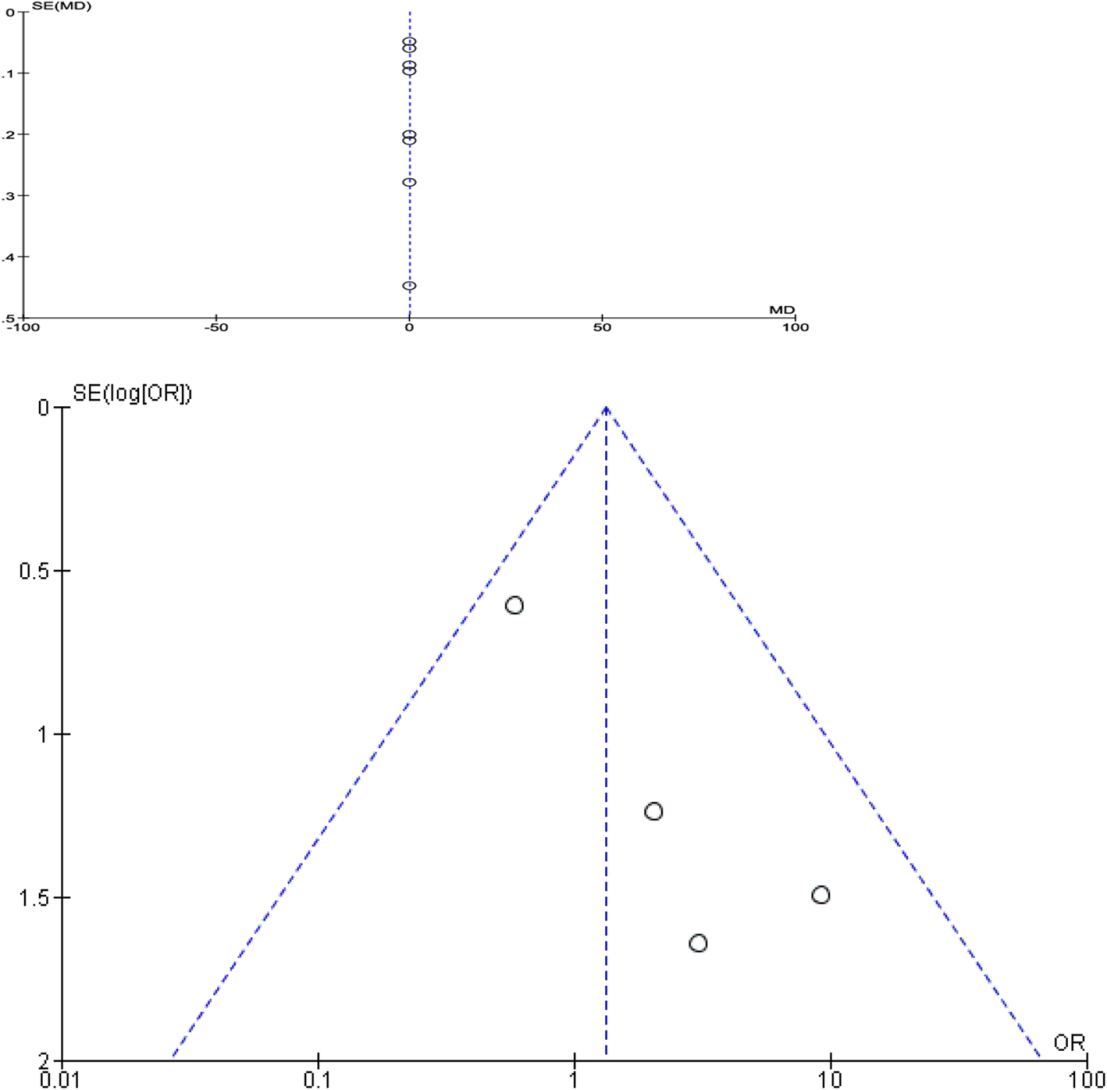
Funnel plot shows APGAR score < 7 at five minutes of norepinephrine versus phenylephrine for treatment of spinal induced hypotension among women undergoing cesarean section: SRMA, 2025

*Apgar Score* < 7 at one minutes: The Apgar score was measured to analyse the effect of two drugs (norepinephrine or phenylephrine) < 7 at 1 min and at 5 min. Both prophylactic (MD = 0.17.95% CI: −0.06–0.4, *p* = 0.15) and therapeutic (MD = 0.03,95% CI: −0.18–0.23, *p* = 0.79) administration have no statistically significant difference in Apgar at 1 min (Fig. 4). However, norepinephrine has a slightly higher Apgar than phenylephrine when administered therapeutically than prophylactically at 5 min (MD = 0.16,95% CI:0.01–0.31, *p* = 0.04) (Fig. 5). In addition, no statistically significant difference was observed in Apgar score at 1 min and five minutes between bolus or infusion administration (Table 3). Egger’s test and funnel plot analysis showed no evidence of publication bias (Figs. 6 and 7). Overall, these findings indicate that neonate born to women treated with norepinephrine for spinal induced hypotension had Apgar score comparable to those treated with phenylephrine, with a slight improvement in 5 min Apgar score during therapeutic administration.

### Neonatal umbilical PCO_2_

**Fig. 8:**
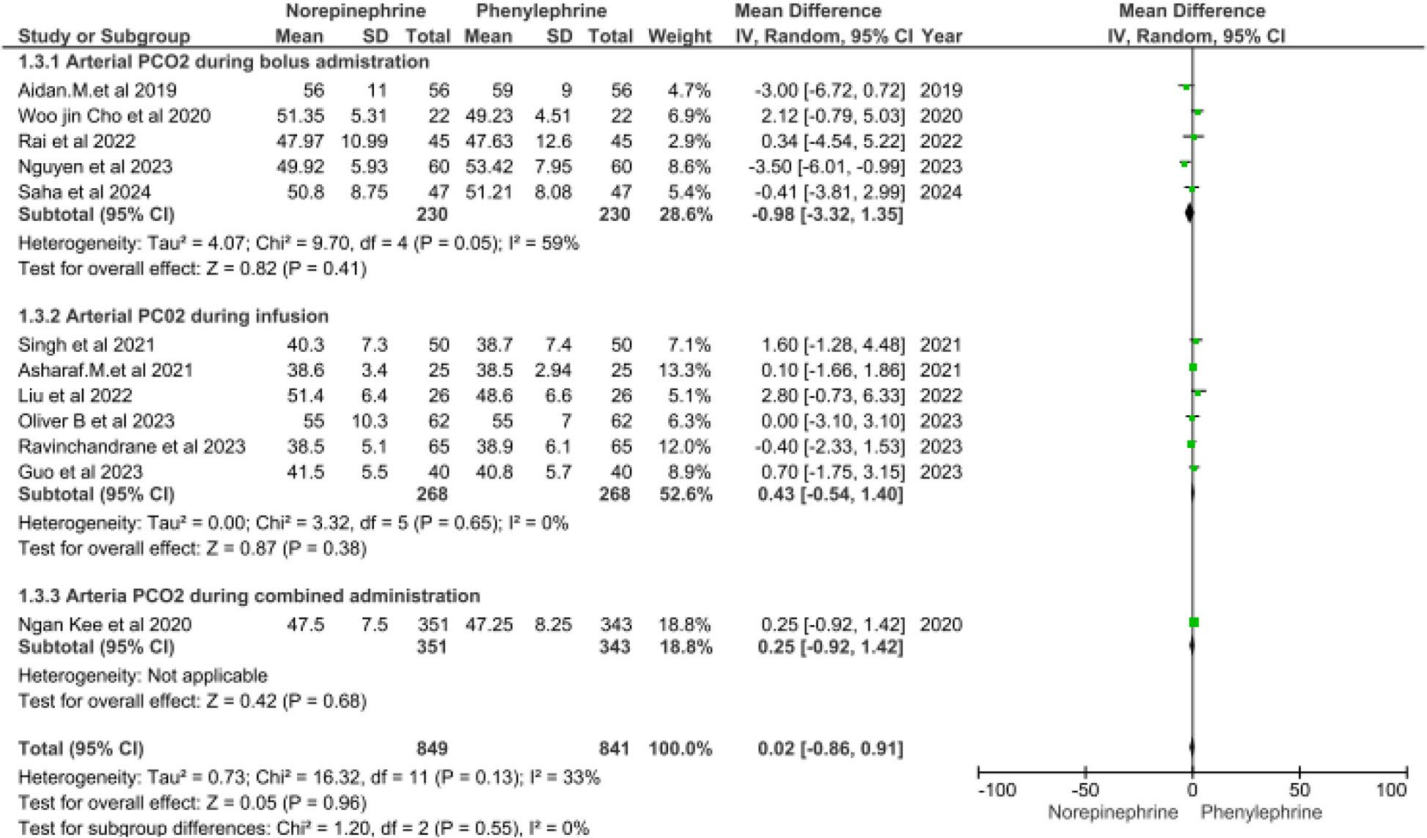
Forest plot shows the subgroup analysis of bolus and infusion on UPCO_2_ following norepinephrine versus phenylephrine for treatment of spinal induced hypotension among women undergoing cesarean section: SRMA, 2025

The subgroup analysis was conducted regarding to methods of administration and dose response on both umbilical arterial partial carbon dioxide and umbilical venous partial carbon dioxide. The pooled effects revealed that norepinephrine is comparable to phenylephrine on UAPCO_2_ when administered either therapeutically, prophylactically, or both (Table 3). Similarly, no statistical significant difference of norepinephrine versus phenylephrine when administered as a bolus, infusion or combined bolus and infusion MD = −0.98, 95% CI: −3.32–1.35, *p* = 0.41, MD = 0.43,95% CI: −0.54–1.4, *p* = 0.38 and MD = 0.25 (−0.42–1.92) respectively (Fig. 8). There is moderate heterogeneity (I^2^ = 59%) in bolus subgroup analysis and no heterogeneity detected (I^2^ = 0%) in infusion subgroup analysis. The amount of dose (Low or high bolus, low and high infusion) of norepinephrine is not statistically significant different from phenylephrine on UAPCO_2_.

Bolus or infusion administration of norepinephrine is not statistically significant different from phenylephrine on UVPCO_2_ but it reduces at low bolus dose administration than a high bolus dose administration (MD = −2.84,95% CI: −5.18- −0.5, *p* = 0.02). Additionally, norepinephrine is reduced UVPCO_2_ when administered therapeutically rather than prophylactically and combined therapeutically and prophylactically (MD = −2.46,95% CI: −4.52- −0.4, *p* = 0.02). No heterogeneity is detected (I^2^ = 0%) (Table 3).

**Fig. 9:**
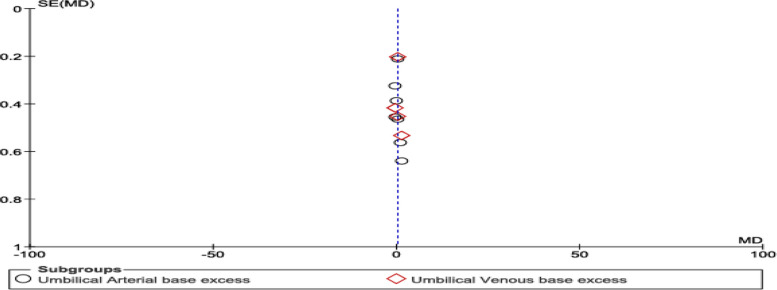
Funnel plot shows base excess of neonatal outcome following norepinephrine versus phenylephrine for treatment of spinal induced hypotension among women undergoing cesarean section: SRMA, 2025

*Neonatal base excess*: The pooled effects demonstrated that norepinephrine has no statistically significant difference on umbilical arterial base excess compared with phenylephrine when administered either therapeutically, prophylactically, both therapeutically and prophylactically, as well as bolus, infusion, and according to infusion dose. Egger’s test is significant (*P* < 0.05) and funnel plot symmetry shows the publication bias among study group is not suspected in umbilical base excess (Fig. 9). However, combined both therapeutic and prophylactic administration of norepinephrine was associated with a significantly higher umbilical venous base excess compared with therapeutic, or prophylactic alone (MD = 1.5,95% CI:0.45–2.55, *p* = 0.005) (Table 3).

### Neonatal Umblical arterial bicarbonate level

**Fig. 10:**
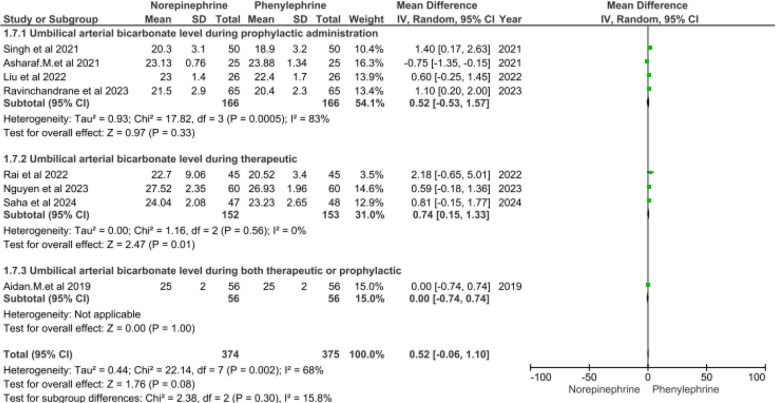
Forest plot shows the subgroup analysis of comparison of prophylactic versus therapeutic administration on UAHCO_3_ following norepinephrine versus phenylephrine for treatment of spinal induced hypotension among women undergoing cesarean section: SRMA, 2025

**Fig. 11:**
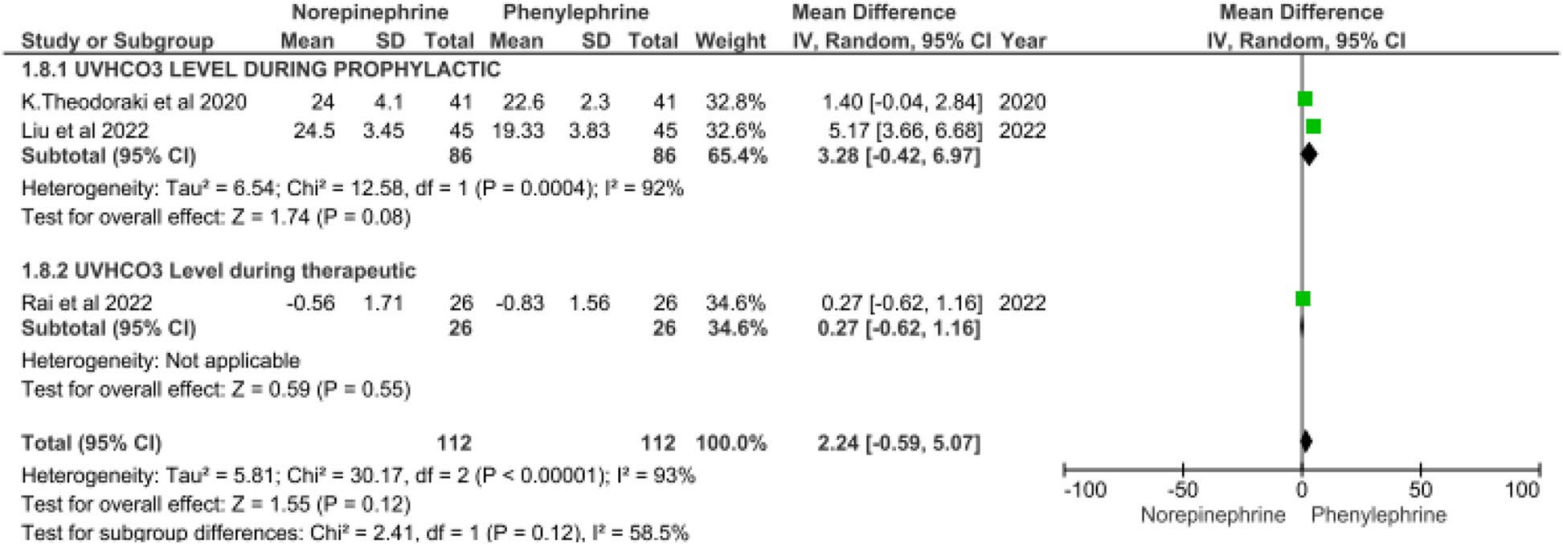
Forest plot shows subgroup analysis comparison of prophylactic versus therapeutic on UVHCO_3_ following norepinephrine or phenylephrine for treatment of spinal induced hypotension among women undergoing cesarean section: SRMA, 2025

The subgroup analysis demonstrated that there is no statistically subgroup difference in UAHCO_3_ level during bolus versus infusion administration (Chi^2^ = 0.00, df = 1, *p* = 0.84, I^2^ = 0%) and during therapeutic and prophylactic administration (Chi^2^ = 2.38, df = 2,*p* = 0.3, I^2^ = 15.8%).According to this analysis norepinephrine increased UAHCO_3_ level during therapeutic adminstration compared with phenylephrine when administerd as prophylactic or combined prophylactic or therapeutic administration (MD = 0.74,95% CI:0.15–1.33, *p* = 0.01) (Fig. 10).Nevertheless,bous versus infusion adminstration, aswell as high versus low dose bolus administration of norepinephrine showed no statistically significant difference compared with phenylephrine (Table 3).Similarly, prophylactic (MD = 3.28,95% CI:−0.42–6.97,*p* = 0.08) and therapeutic administration (MD = 0.27,95% CI:−0.62–1.16, *p* = 0.55 (Fig. 11) of norepinephrine is statistically similar effect on UVHCO_3_. However, due to the high heterogenity among subgroup anlaysis,the certainity of evidence may be downgraded. Additionally, bolus or infusion administration of norepinephrine showed no statistically significant difference compared with phenylephrine regarding UVHCO_3._

**Fig. 12:**
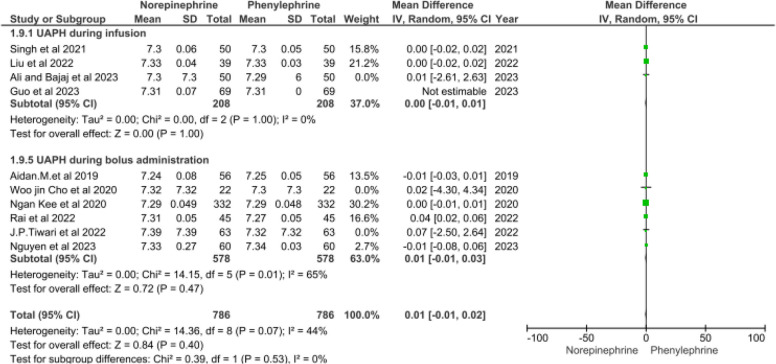
Subgroup analysis of bolus versus infusion on UAPH in neonatal outcome following norepinephrine versus phenylephrine for treatment of spinal induced hypotension among women undergoing cesarean section: SRMA, 2025

*Neonatal Arterial Umblical PH effect*: from this analysis, there is no statistically significant difference between norepinephrine and phenylephrine on neonatal umbilical arterial PH during bolus (MD = 0.01,95% CI: 0.01–0.03, *p* = 0.47 or infusion (MD = 0.00,95% CI: −0.01–0.01, *p* = 1.0). Norepinephrine has no statistically significant difference from phenylephrine during therapeutic, prophylactic or combined therapeutic or prophylactic (Fig. 12).

### Neonatal umbilical venous PH

**Fig. 13:**
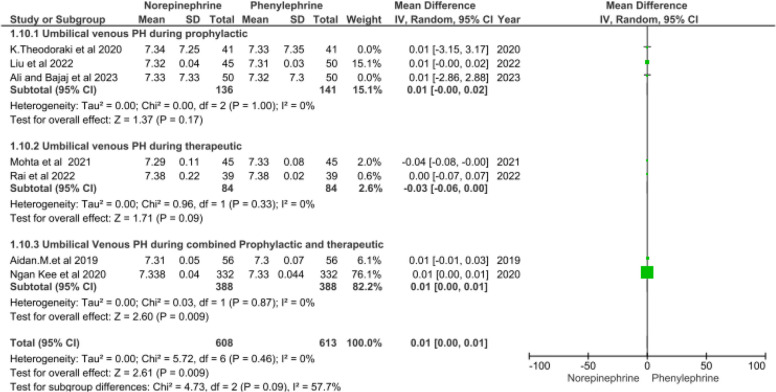
Forest plot shows subgroup analysis of comparison of therapeutic versus prophylactic on venous umbilical PH in neonatal outcome following norepinephrine versus phenylephrine for treatment of spinal induced hypotension among women undergoing cesarean section: SRMA, 2025

The pooled results revealed that combined therapeutic or prophylactic administration of norepinephrine increased umbilical arterial PH statistically more than therapeutic or prophylactic alone (MD = 0.01,95% CI: 0.00–0.01, *p* = 0.009) (Fig. 13). Despite this, there is no statistically significant difference in the administration of norepinephrine with phenylephrine regarding bolus or infusion administration (Table 3).

### Neonatal umbilical arterial PO_2_

**Fig. 14:**
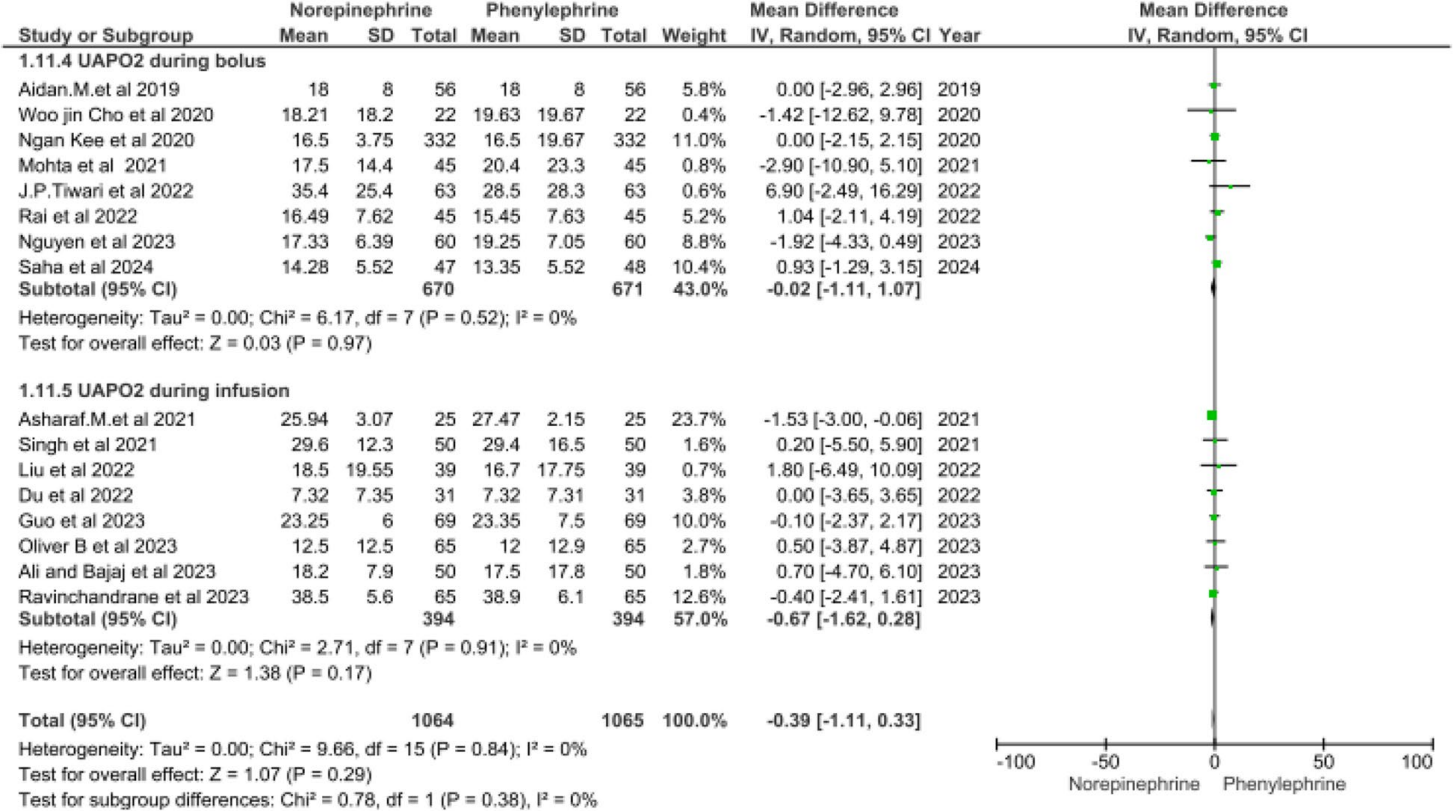
Subgroup analysis of bolus versus infusion for UAPO_2_ in neonatal outcome following norepinephrine versus phenylephrine for treatment of spinal induced hypotension among women undergoing caesarean section: SRMA,2025

Subgroup analysis revealed that norepinephrine has no statistically significant effect with phenylephrine regarding methods of administration, dose (bolus or infusion dose) (Fig. 14), at low or high bolus administrations.

### Haemodynamic characteristics of mothers

**Fig. 15:**
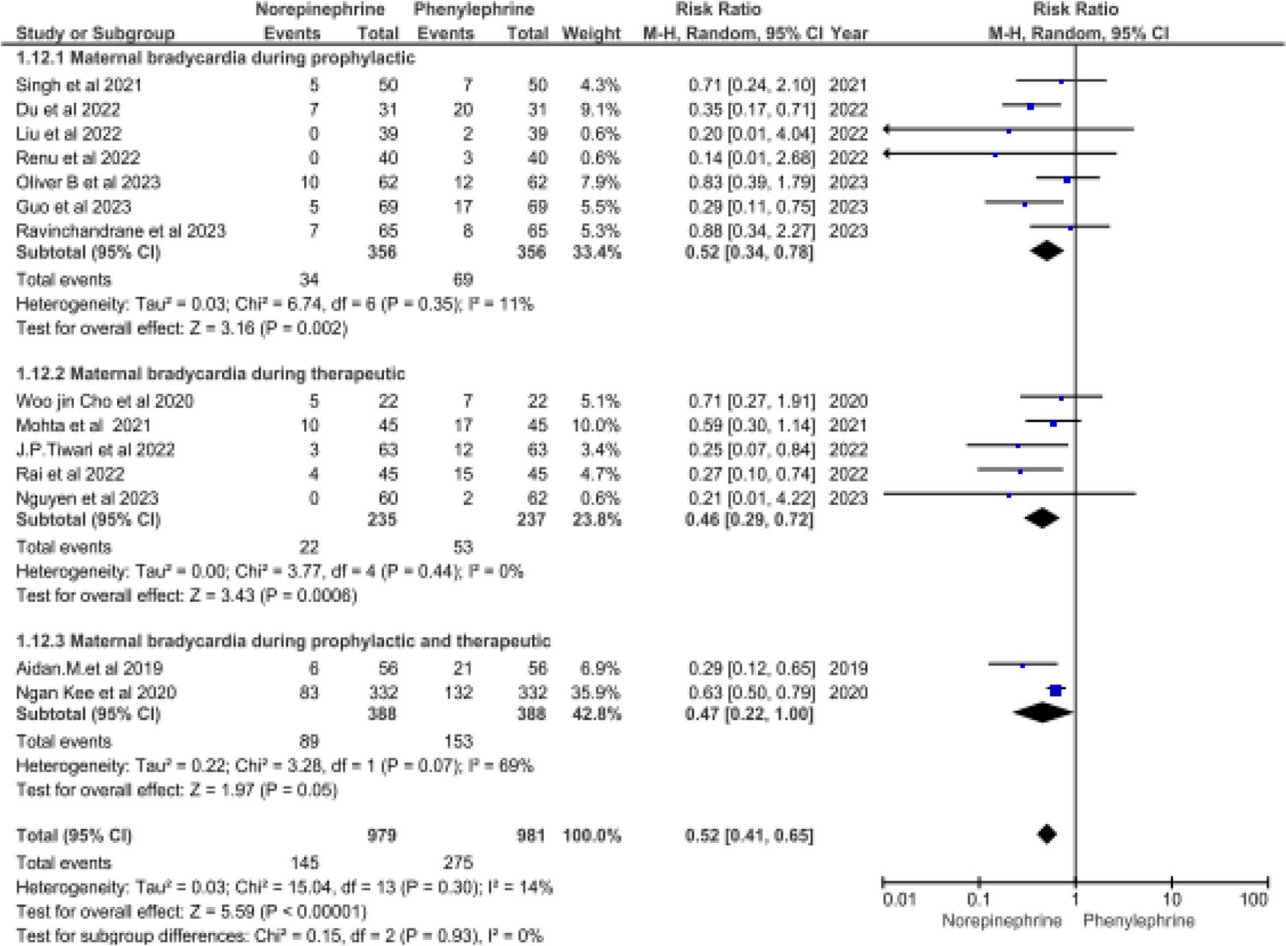
Subgroup analysis shows comparison of prophylactic versus therapeutic on maternal bradycardia following norepinephrine versus phenylephrine for treatment of spinal induced hypotension among woman undergoing cesarean section: SRMA, 2025

*Bardycardia*: Subgroup analysis based on administration demonstrated that norepinephrine decreased maternal bradycardia more than phenylephrine in both prophylactic and therapeutic administration**.** Norepinephrine reduced maternal bradycardia both in prophylactic (RR = 0.52,95% CI:0.34–0.78, *p*−0.02) and in therapeutic group (RR = 0.46, 95% CI:0.29–0.72, *p* = 0.0006) compared with phenylephrine (Fig. 15). Similarly, norepinephrine reduced maternal bradycardia at low bolus doses than higher dose (RR = 0.49,95% CI:0.37–0.72, *p* = 0.0003) and during bolus administrations (RR = 0.49,95% CI:0.35–0.67, *p* < 0.0001 and infusion administration (RR = 0.53,95% CI:0.53–0.81, *p* = 0.003) compared with phenylephrine. Norepinephrine also reduced maternal bradycardia in elective caesarean section compared with emergeny procedures (RR = 0.39,95% CI: 0.29–0.59, *p* < 0.00001) compared with phenylephrine. Regarding pregnancy type both singleton pregnacies (RR = 0.41,95% CI: 0.28–0.59,*p* < 0.00001) and twin pregnacies (RR = 0.35,95% CI:0.17–0.71,*p* = 0.003) had a lower risk of maternal bradycardia with norepinephrine than phenylephrine.

**Fig. 16:**
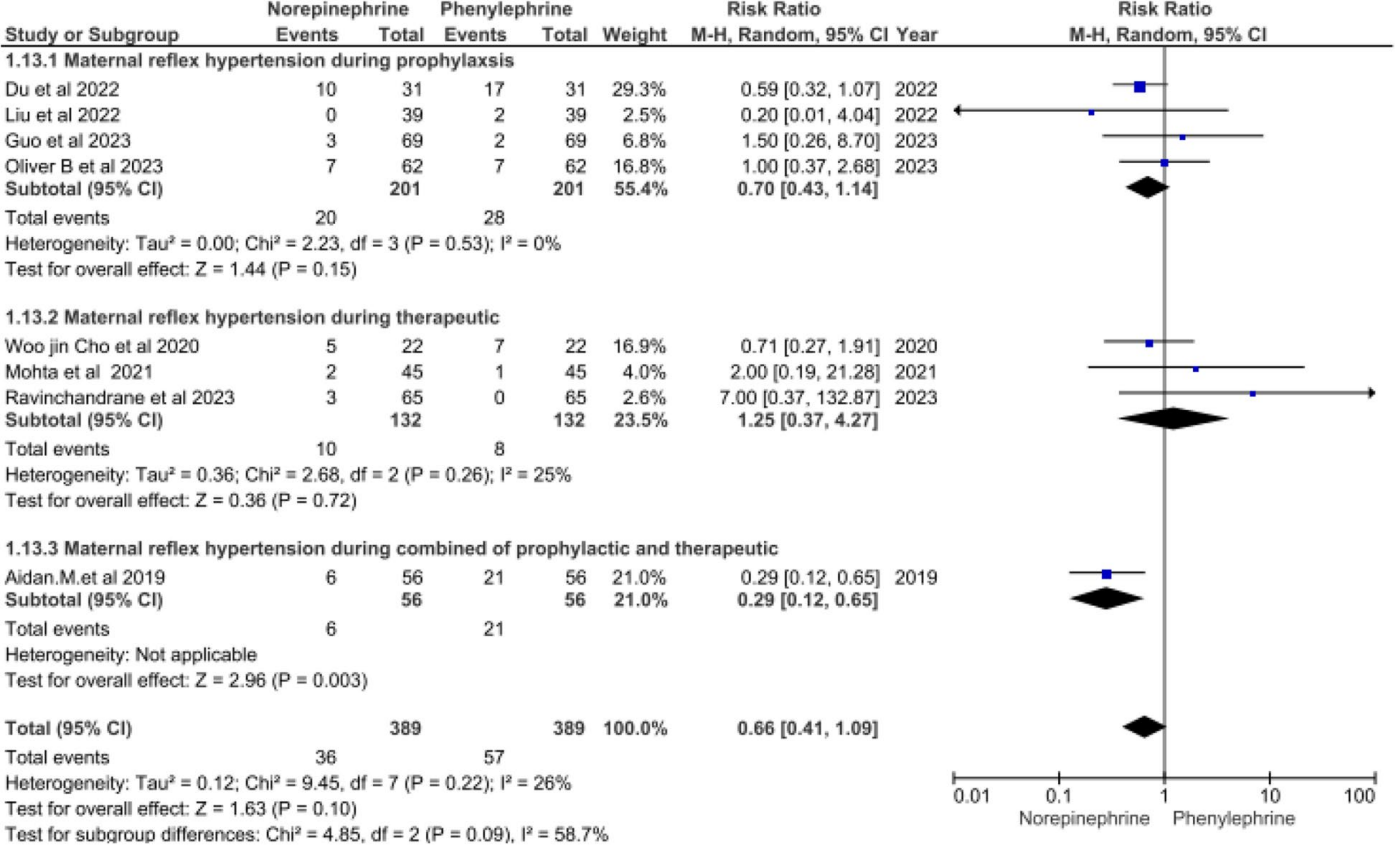
Subgroup analysis showed comparison of therapeutic versus prophylactic effects on maternal reflex hypertension following norepinephrine versus phenylephrine for treatment of spinal induced hypotension among women undergoing caesarean section: SRMA,2025

*Maternal reflex hypertension*: The pooled analysis showed that norepinephrine administration during combined of both prophylactic and therapeutic use was associated lower risk of maternal reflex hypertension compared with phenylephrine (RR = 0.29,95% CI:0.12–0.65, *p* = 0.003) (Fig. 16). However, norepinephrine has no statistically significant difference when administered either bolus or infusion, low bolus dose or high bolus dose, low infusion dose or high infusion dose, for singleton, twin or both and for elective or emergency compared with phenylephrine (Table 3).

### Incidence of hypotension

**Fig. 17:**
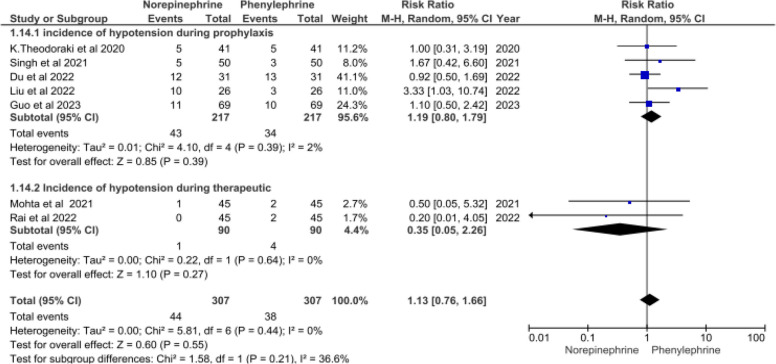
Subgroup analysis showed comparison of therapeutic versus prophylactic effects on incidence of hypotension following norepinephrine versus phenylephrine for treatment of spinal induced hypotension among women undergoing caesarean section: SRMA,2025

**Fig. 18:**
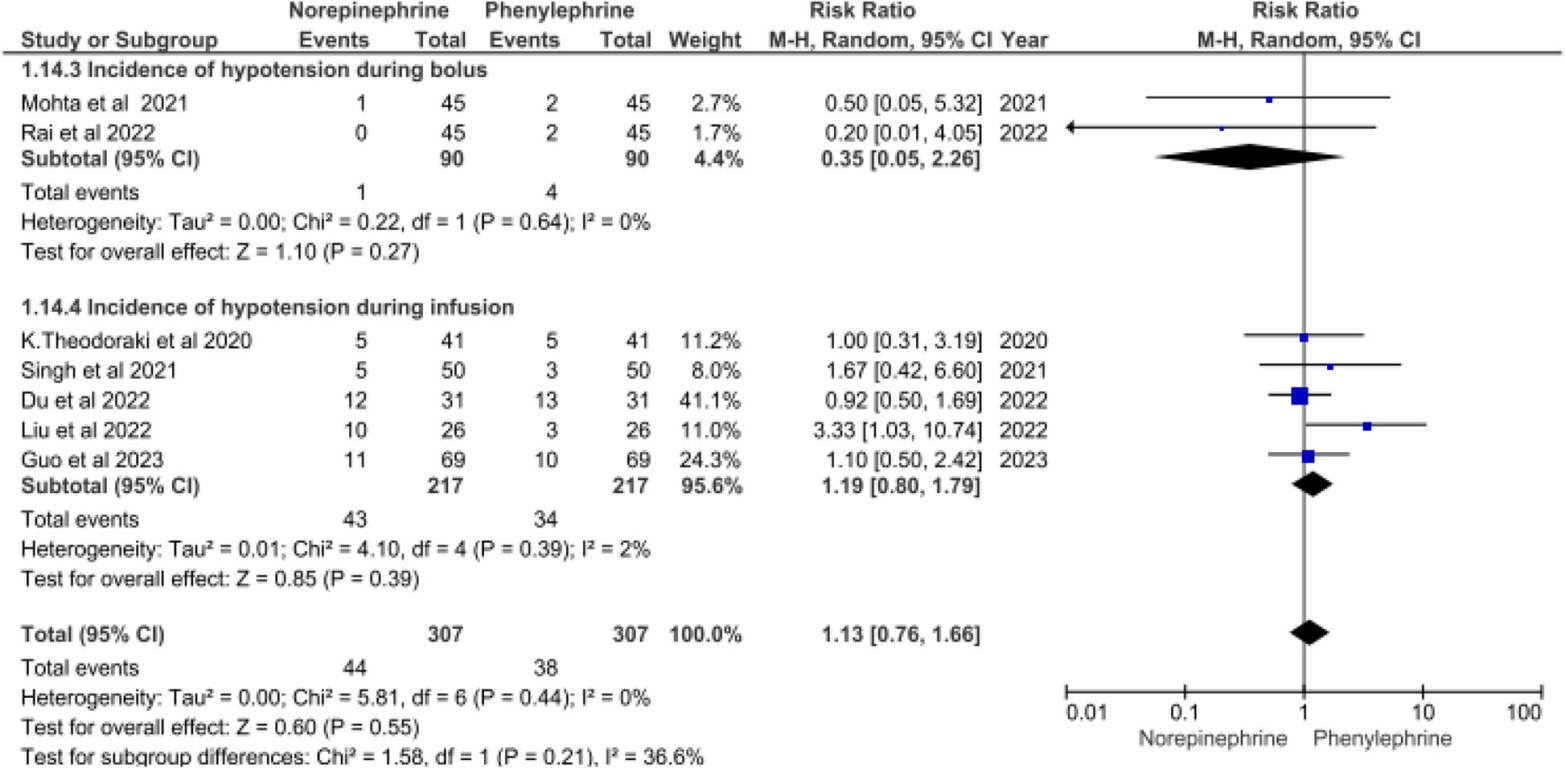
Subgroup analysis shows comparison of bolus versus infusion effects on the incidence hypotension following norepinephrine versus phenylephrine for treatment of spinal induced hypotension among women undergoing caesarean section: SRMA,2025

The incidence of hypotension in parturients who take norepinephrine is not statistically significance difference either therapeutically (RR = 0.35,95% CI:0.05–2.26, *p* = 0.27) or prophylactically administration (RR = 1.19,95% CI:0.8–1.79) when compared with phenylephrine (Fig. 17). Similarly, the incidence of hypotension in norepinephrine is statistically similar to phenylephrine during bolus or infusion (Fig. 18).

## Discussion

This systematic review and meta-analysis is providing comprehensive information on neonatal outcome and maternal haemodynamics effects associated with norepinephrine and phenylephrine for the prevention and treatment of spinal induced hypotension. The findings insights into the comparative effect of those medications on neonatal outcomes, such as Apgar score, umbilical PH, umbilical PO_2_, umbilical PCO_2_, bicarbonate level, base excess, maternal bradycardia, reflex hypertension and incidence of hypotension.

The pooled results showed that there were comparable Apgar score < 7 at one minutes between norepinephrine and phenylephrine during therapeutic or prophylactic administration. However, norepinephrine had higher Apgar score less than seven at five minutes during therapeutic administration than prophylactic compared with phenylephrine. Norepinephrine had no meaningful difference on Apgar score at one minute and five minutes compared with phenylephrine when administered as bolus or infusion. This is consistent with the review by Hessen et al., showed that norepinephrine had no significant difference on Apgar score at 1 and 5 min compared with phenylephrine (Heesen et al. 2020).

The finding demonstrated that norepinephrine is similar to phenylephrine regarding to UAPH whether administered as therapeutic, prophylactic or combined. There is also no significant difference of norepinephrine compared to phenylephrine during bolus or infusion administration. Our finding is concordance with systematic review and meta-analysis done by Signa vitae et al. which showed that norepinephrine does not increase neonatal foetal acidosis more than phenylephrine (Song 2024). Combined therapeutic and prophylactic administration of norepinephrine has increased UVPH than prophylactic or therapeutic administration compared with phenylephrine. Norepinephrine at low bolus dose has increased UVPH than high bolus dose compared with phenylephrine. But no statistically significant difference of norepinephrine compared with phenylephrine during bolus or infusion administration.

In our review and meta-analysis, norepinephrine has similar UAPCO_2_ and UAPO_2_compared with phenylephrine, whether administered bolus or infusion, therapeutic or prophylactic. This is in line with study by Guo et al. (Guo et al. 2022), Putheenvettil et al. (Article 2021), and Vishnu Narayan et al. (Vnm and Article 2024) which reported no significant difference in neonatal UPCO_2_ and UAPO_2_during prophylactic(bolus) and therapeutic (infusion) administration. A possible explanation is that both norepinephrine and phenylephrine are improve placenta blood flow, oxygen supply, decreasing metabolic level, and minimal cross placenta (Feng 2020). However, subgroup analysis showed that therapeutic administration of norepinephrine reduced UVPCO_2_ than prophylactic compared with phenylephrine. Similarly, low dose bolus administration of norepinephrine was associated with lower UVPCO_2_ than high dose bolus administration relative to phenylephrine. The sensitivity analysis demonstrated that removing of single study changes the overall pooled effect for UVPCO_2_ during bolus administration of norepinephrine compared with phenylephrine. Even though, similarity of norepinephrine with phenylephrine on UVPCO_2_, the results require caution during using it.

A randomised controlled trials by Liu et al., Woo jin et al.,Ngaan kee et al., Saha et al., Oliver B et al., and Aidan.M et al. (Sharkey et al. 2019; Cho 2020; Kee et al. 2020; Belin et al. 2023; Article 2024; Wang 2018) showed that norepinephrine and phenylephrine are comparable regarding to base excess. Similarly, our pooled results demonstrated that norepinephrine has no statistically significant effect on umbilical arterial base excess when administered as prophylactic, therapeutic, combined therapeutic and prophylactic, bolus or infusion and at high infusion dose or low infusion dose compared with phenylephrine. Apart from this, combined (therapeutic and prophylactic administration) of norepinephrine increases venous base excess than prophylactic or therapeutic alone compared with phenylephrine.

Our review and meta-analysis demonstrated that there is no statistically significant difference between norepinephrine and phenylephrine on UAHCO_3_ level when administrated as a bolus or infusion. This study is in line with study by Saha et al.which reported no significant difference of UAHCO_3_level during bolus regimen of norepinephrine and phenylephrine but contradicted with studies by Mohta et al. (Mohta et al. 2019) showed that norepinephrine is produces more metabolic acidosis than phenylephrine and Singh et al. (Singh et al. 2022) phenylephrine is produces more metabolic acidosis than norepinephrine. Additionally, study by K.Theodoreki et al. (Theodoraki et al. 2020) showed that Phenylephrine is produces more metabolic alkalosis than norepinephrine while Ravichandrane et al. (Randomized 2023) revealed that norepinephrine is produce more metabolic alkalosis than phenylephrine. Therapeutic administration of norepinephrine is increases UAHCO_3_ more than prophylactic and combined prophylactic and prophylactic administration compared with phenylephrine. Norepinephrine has no statistically significant difference from phenylephrine, whether administered as bolus or infusion, therapeutic and prophylactic compared with phenylephrine on UVHCO_3_.

The pooled results revealed that norepinephrine reduced bradycardia compared with phenylephrine when administered as either bolus and infusion; however, low bolus dose administration showed a greater reduction in bradycardia than high bolus dose. Regarding timing of administration, both prophylactic and therapeutic administration of norepinephrine reduced maternal bradycardia compared with phenylephrine. This study is in line with studies by, Sharkey. AM et al., Xu et al. and Hasanin et al. (Sharkey et al. 2019; Xu et al. 2018; Hasanin et al. 2019) which reported that parturients receiving norepinephrine experienced a lower incidence of bradycardia than those receiving phenylephrine. Our results also reported that elective caesarean section patients who received norepinephrine had a lower risk of bradycardia than emergency caesarean section compared to phenylephrine. Similarly, singleton pregnancies were associated with a lower risk of bradycardia than twin pregnancies who received norepinephrine than phenylephrine.

The analysis on maternal reflex hypertension reported that norepinephrine had no statistically meaningful difference with phenylephrine when administered as a bolus or infusion, and at low bolus or infusion. This study is concordance with meta-analysis by Song. J et al. (Song 2024) which reported the norepinephrine and phenylephrine produced similar incidence of reflex hypertension. However, combined therapeutic and prophylactic administration of norepinephrine appeared to be associated with a lower risk less risk of maternal reflex hypertension compared with prophylactic or therapeutic alone. The incidence of hypotension in parturients receiving norepinephrine was no statistically significant difference regardless of timing of administration and dose response compared with phenylephrine.

### The strength of the review and meta-analysis

This review included a recent relevant study related to the neonatal outcome and maternal outcome. 1.All of the studies were randomized controlled trials. 2.The risk of bias and level of certainty were checked. 3. The analysis include timing of administration (therapeutic or prophylaxis), dose response bolus or infusion.4. The review contains trials of multiple countries.

Limitation: The analysis has the following limitations.

- 1. The studies with a high risk of bias and low certainty were included.
- 2. The studies that have continuous variable studies with medians were taken as means.
- 3. Variables definitions of parameters.
- 4. Variety of sample size included may leads to publication bias and downgrade certainty of evidence.
- 5. High risk patients (severe preeclampsia, cardiac patients and other chronic disease) are not considered.
- 6. The majority of included case were elective so, this analysis limited for generalizability for all patients.

## Conclusion

This systematic review and meta-analysis shows women treated with bolus (dose range 4mcg/ml-10 mg/ml) of norepinephrine versus (50mcg/ml-100mcg/ml) of phenylephrine and infusion (fixed rate dose range 4mcg/min-6mcg/min for norepinephrine and 4mcg/min-25mcg/min for phenylephrine) have comparable neonatal and maternal haemodynamic effects. Similarly, weight based adjusted infusion dose range (0.05mcg/kg/min-0.08mcg/kg/min for norepinephrine and 0.1mcg/kg/min-0.625mcg/kg/min for phenylephrine) and prophylactic or therapeutic administration of norepinephrine have comparable neonatal outcome and maternal haemodynamics with phenylephrine. Therefore, norepinephrine is an alternative vasopressor for management of spinal induced hypotension and for good neonatal outcome.

## Supplementary Information

Additional file 1. Table S1:Searching methods by PubMed. Table S2: Searching methods by Cochrane library. Table S3: Summary of sensitivity analysis for neonatal outcome following norepinephrine versus phenylephrine for treatment of spinal-induced hypotension among women undergoing cesarean section: systematic review and meta-analysis of randomized controlled trials

## Data Availability

No datasets were generated or analysed during the current study.

## Abbreviations

APGAR: Appreance, pulse, grimace, activity, respiration
CI: Confidence interval
HCO3: Bicarbonate
GRADE: Grade of recommendation, assessment, development and evaluation
MD: Mean difference
PCO2: Partial carbondiaxodie
RCT: Randomiszed controlled trial
PICO: Population,intervention,comparatives and outcome
PO2: Partial oxygen
SRMA: Systematic review and meta-analysis
UA: Umbilical artery
UAPCO2: Umbilical arterial partial carbondioxide
UAPO2: Umbilical arterial partial oxygen
UV: Umbilical vein
UVPCO2: Umbilical venous partial carbondioxide
UVPO2: Umbilical venous partial oxygen
