# Supplementary material for: Neonatal outcome following norepinephrine versus phenylephrine for treatment of spinal-induced hypotension among women undergoing caesarean section: systematic review and meta-analysis of randomised controlled trials
Source: Perioper Med (Lond). 2026 May 28;15:72. doi: 10.1186/s13741-026-00701-5 (PMC13483524; doi:10.1186/s13741-026-00701-5)
Supplement: Supplementary file 1 [file 13741_2026_701_MOESM1_ESM.docx]

Supplementary material

Table S1:Searching methods by PubMed

| Searching methods | Yields |
| --- | --- |
| Neonatal (All fields) “OR” newborn (All fields) | 7541 |
| Neonatal outcome (MeSH terms) “AND” Phenylephrine (MeSH terms) ‘OR Vasopressor(MeSH terms) | 376 |
| Neonatal outcome (MeSH terms) “AND” Phenylephrine (MeSH terms) “OR” Vasopressor (MeSH terms) | 351 |
| Spinal induced hypotension (MeSH terms) ‘OR’ lower systolic blood pressure (MeSH terms) “AND” Caesarean Section (MeSH terms) | 29 |
| Spinal anesthesia induced hypotension (All fields) ‘OR’ lower systolic blood pressure (MeSH terms) ‘AND’ Caesarean Section (All fields) | 201 |
| Norepinephrine (All fields)” OR” alpha 1-adnergic agonist and Beta-adrenergic agonist (All fields) “AND” Phenylephrine (All fields) “OR” selective alpha -1 agonist “AND” spinal anesthesia (all fields) | 44 |
| Norepinephrine (all fields) “AND” Phenylephrine (All fields) “AND” Neonatal outcome (all fields) “AND” Cesarean section (all fields) | 18 |
| Norepinephrine (all fields) “OR” α and β adrenergic agonists (All fields)”AND” Phenylephrine “OR” selective alpha 1 agonist (All fields) “AND” Spinal hypotension “AND” Prevention “OR” treatment | 828 |
| Neonatal outcome (All fields) “AND” Phenylephrine “OR” selective alpha 1 and beta adrenergic agonist (All fields) “AND” Spinal hypotension (All fields) “AND” Prevention (All fields) | 15 |
| Norepinephrine (MeSH terms) “OR” α and β adrenergic agonists (MeSH terms)”AND” Phenylephrine “OR” selective alpha 1 agonist (MeSH terms) “AND” Spinal hypotension (MeSH terms)“AND” Prevention (MeSH terms)“OR” treatment (MeSH terms) | 0 |

**Table S2: Searching methods by Cochrane library**

| No | Boolean operator | Searching methods | Mesh terms | Results |
| --- | --- | --- | --- | --- |
| #1 | “AND” | Neonatal (Abstract/title) | limits | 75 |
| #2 | “OR” | new born (Abstract/title) | limits | 3199 |
| #3 | “AND” | Norepinephrine (Abstract/title) | limits | 8639 |
| #4 | “OR” | alpha 1and beta adrenergic agonist (Abstract/title) | limits | 296 |
| #5 | “AND” | phenylephrine | limits | 2850 |
| #6 | “OR” | Selective alpha 1 agonist (Abstract/title) | limits | 305 |
| #7 | “AND” | Spinal induced hypotension (Abstract/title) | limits | 845 |
| #8 | “OR” | Lower systolic blood pressure (Abstract/title) | limits | 12478 |
| #9 | “AND” | Caesarean section (Abstract/title) | limits | 18589 |
| #10 | “AND” | #9 & #5 | limits | 27956 |
| #11 | “AND” | #3&#7 | limits | 5789 |
| #12 | “AND” | #10&#6 | limits | 29 |
| #13 | “AND” | #1,#8&7 | limits | 236 |
| #14 | “AND” | #4,#10,#11,&#12 | limits | 783 |
| #15 | “AND” | #13&14 | limits | 333 |

Table S3: Summary of sensitivity analysis for neonatal outcome following norepinephrine versus phenylephrine for treatment of spinal-induced hypotension among women undergoing cesarean section: systematic review and meta-analysis of randomized controlled trials

| Comparison group | Sample size | Effect size | CI with 95% | P-Value | I^2^ (%) |
| --- | --- | --- | --- | --- | --- |
|  |  |  |  |  |  |
| Umbilical venous partial carbon dioxide (UVPCO2) during bolus administration with infusion | 252 | MD= -2.46 | -4.52- -0.4 | 0.02 | 0 |
| Umbilical arterial base excess during prophylactic administration with therapeutic | 445 | MD= -0.37 | -0.88-0.15 | 0.16 | 0 |
| Umbilical arterial base excess during infusion with bolus | 364 | MD= -0.37 | -0.88 -0.15 | 0.16 | 0 |
| Umbilical arterial bicarbonate level during prophylactic administration | 388 | MD=0.95 | 0.4-1.5 | 0.0008 | 0 |
| Umbilical arterial PH during therapeutic administration comparison with prophylactic | 592 | MD=0.04 | 0.02-0.06 | 0.004 | 0 |
| Umbilical arterial PH during bolus administration with infusion | 1268 | MD= -0.00 | -0.01-0.01 | 0.8 | 0 |
| Maternal reflex hypertension during bolus administration with infusion | 268 | RR=0.83 | 0.34-2.06 | 0.69 | 0 |
